# Synergy between Defects and Lattice Distortion Drives Self‐Powered Elastico‐Near‐Infrared Mechanoluminescence in Cr^3+^‐Doped Spinel Oxides

**DOI:** 10.1002/advs.202510848

**Published:** 2025-08-07

**Authors:** Yao Xiao, Kang Chen, Mingzi Sun, Puxian Xiong, Bolong Huang, Yongsheng Sun, Dongdan Chen, Jiulin Gan, Zhongmin Yang

**Affiliations:** ^1^ State Key Laboratory of Luminescent Materials and Devices, Institute of Optical Communication Materials; Guangdong Engineering Technology Research and Development Center of Special Optical Fiber Materials and Devices, Guangdong Provincial Key Laboratory of Fiber Laser Materials and Applied Techniques South China University of Technology Guangzhou 510640 China; ^2^ Department of Chemistry City University of Hong Kong Tat Chee Avenue Kowloon Hong Kong SAR 999077 China; ^3^ Department of Electrical and Electronic Engineering The University of Hong Kong Pok Fu Lam Rd Hong Kong Island Hong Kong SAR 999077 China; ^4^ Research Institute of Future Technology South China Normal University Guangzhou 510631 China

**Keywords:** intrinsic defects, lattice distortion, near‐infrared mechanoluminescence, self‐powered, synergy effect

## Abstract

Elastico‐mechanoluminescence (ML) enables unique force‐to‐light transduction for applications in human‐machine interaction and smart sensing, yet traditional trap‐controlled models fail to explain self‐powered ML phenomena. Here, a Cr^3+^‐doped spinel oxide exhibiting autonomous near‐infrared (NIR) ML is reported, where self‐powered emission originates from synergistic interactions between local lattice distortions and multi‐defect networks. Theoretical calculations reveal that Cr^3+^ doping activates nearest‐neighbor sites to generate mid‐gap states, facilitating stress‐driven electron tunneling to luminescent centers without external excitation. The material shows narrowband NIR emission (711 nm) from the spin‐forbidden transition, with linear ML intensity response to mechanical stress and negligible persistent luminescence. Proof‐of‐concept demonstrations in bright‐field anti‐counterfeiting (NIR QR‐code imaging) and biomedical tissue penetration (10 mm pork) validate its practical utility. This work establishes a defect‐distortion coupling mechanism for self‐powered NIR‐ML, providing a theoretical framework to guide the design of next‐generation autonomous optomechanical materials for energy‐efficient sensing and bio‐imaging.

## Graphical abstract

This work demonstrates crystal lattice tailoring and defect modulation to engineer a Cr^3+^‐activated cubic spinel oxide, where strategic introduction of Cr^3+^ into octahedral Al^3+^/Li^+^ sites drives efficient NIR‐ML through a non‐trap‐based mechanism.

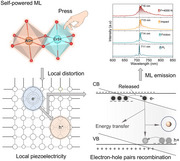

## 1 Introduction

Elastico‐mechanoluminescence (ML) represents a cutting‐edge mechanical‐to‐optical transduction phenomenon, wherein materials emit light upon mechanical stimulation‐including deformation, compression, or flexure—without external energy input.^[^ ^1^, ^2^ ^]^ Distinct from conventional luminescence modes, ML uniquely harvests ambient mechanical energy, offering a sustainable pathway for real‐time energy conversion with applications spanning intelligent sensing,^[^ ^3^ ^]^ secure data encoding, and biomedical diagnostics.^[^ ^4^ ^]^ Since the landmark discovery of SrAl_2_O_4_:Eu^2+^ and ZnS:Mn^2+^ by Xu et al. in 1998, ML materials have expanded across the electromagnetic spectrum, yet progress remains hindered by an incomplete theoretical framework.^[^ ^5^, ^6^ ^]^ Current material development relies heavily on empirical trial‐and‐error, as the intricate relationship between defect structures, lattice dynamics, and luminescent responses remains poorly understood. This knowledge gap limits the rational design of next‐generation ML systems optimized for practical scenarios, such as wearable health monitors or adaptive security labels.^[^ ^7^ ^]^ Addressing this challenge requires a systematic exploration of mechanoluminescent mechanisms‐decoding how atomic‐scale defects and mechanical stress synergistically regulate charge carrier behavior to enable efficient light emission. By advancing mechanistic understanding beyond descriptive models, researchers can unlock predictive design strategies, accelerating the discovery of high‐performance ML materials with tailored spectral properties and mechanical robustness. Such advancements are pivotal for translating ML from laboratory curiosity to transformative technology in autonomous sensing and energy‐efficient optoelectronics.

ML materials are commonly classified into self‐powered (non‐pre‐irradiated) and trap‐controllable (pre‐irradiated) types based on their luminescence mechanisms and performance.^[^ ^8^ ^]^ Trap‐controllable ML relies on carrier recombination in defect traps with specific depths (0.60–0.75 eV),^[^ ^9^ ^]^ where mechanical stress releases trapped electrons to recombine with holes at luminescent centers.^[^ ^10^, ^11^, ^12^ ^]^ However, this mechanism faces critical limitations: external excitation sources increase application costs and compromise sensing accuracy, potential phototoxicity restricts in vivo biomedical use, and persistent luminescence (PersL) causes low‐contrast images and weak signals. In contrast, self‐powered ML materials operate without external excitation, offering stable recoverability and thermal stability.^[^ ^13^, ^14^ ^]^ For example, ZnS:Cu^2+^/polymer composites exhibit excellent cyclic ML stability, while Pr^3+^/Tb^3+^‐doped calcium niobates achieve tunable ML behavior via lattice engineering.^[^ ^15^, ^16^ ^]^ Systems like Lu_3_Al_5_O_12_:Ce^3+^/PDMS and CaF_2_:Tb^3+^ elastomers demonstrate efficient self‐powered ML through triboelectric effects, validated by contact electrification theory.^[^ ^17^, ^18^, ^19^, ^20^, ^21^ ^]^ Organic‐inorganic interface systems and ZnS‐based heterojunctions have also enabled self‐powered ML mechanisms.^[^ ^22^, ^23^ ^]^ Despite these advances, most self‐powered ML materials are limited to Mn^2+^‐activated visible emitters in sulfur/fluoride matrices, suffering from poor environmental compatibility. Developing oxide‐based near‐infrared (NIR) ML materials, such as Cr^3+^/Nd^3+^‐doped spinel structures, holds promise for bio‐detection, anti‐counterfeiting, and deep‐tissue imaging, addressing both spectral specificity and biocompatibility challenges in autonomous sensing technologies.^[^ ^24^, ^25^ ^]^

**Figure 1:**
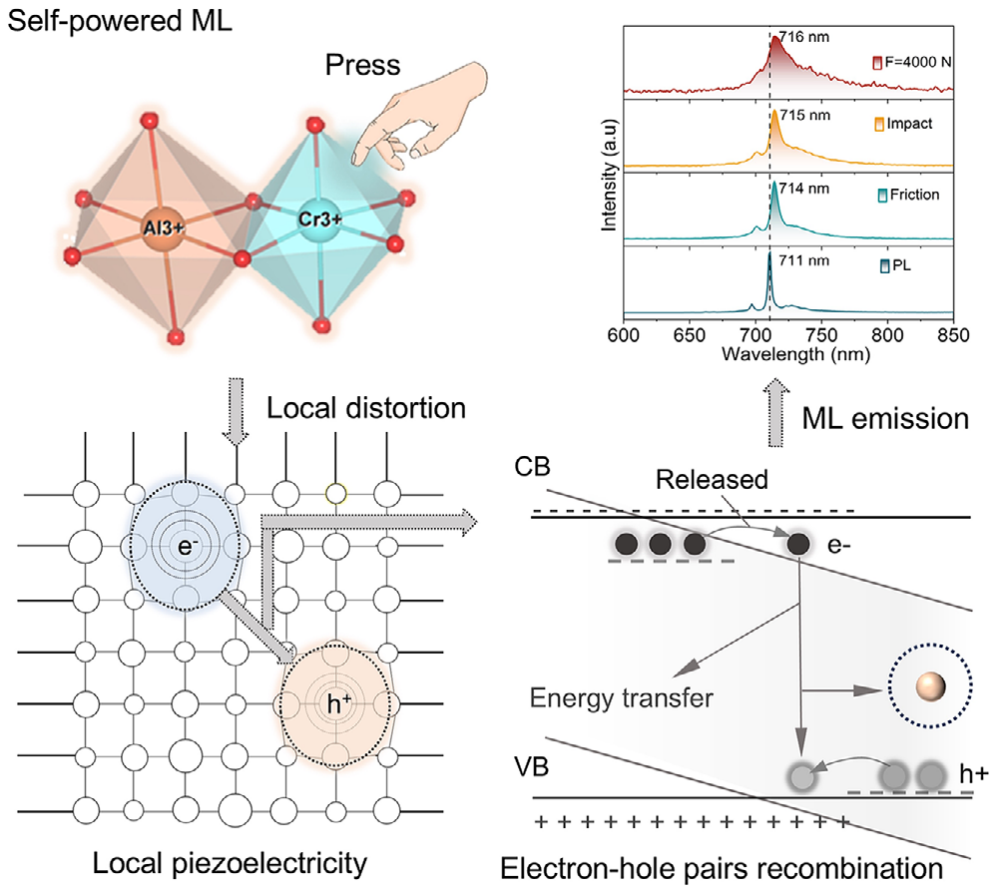
Schematic of defect and local distortion induced self‐powered ML mechanism. The PL and ML spectra of LiAl_4.99_O_8_: 0.01Cr^3+^ under different excitation conditions (Xe lamp for 254 nm, friction, impact, stress).

In the self‐powered ML materials, the dominance of Mn^2+^‐activated visible emitters contrasts sharply with the underexplored potential of Cr^3+^, a transition metal ion capable of enabling near‐infrared (NIR) emissions with superior tissue penetration and anti‐counterfeiting utility. While Mn^2+^‐based systems are limited to visible spectral ranges and suffer from environmental instability, Cr^3+^‐doped materials offer a transformative pathway for NIR‐ML, yet their mechanistic understanding remains fragmented, with prior reports largely restricting ML activity to repetitive rubbing in elastic hosts.^[^ ^26^, ^27^ ^]^ Following our previous study,^[^ ^28^, ^29^ ^]^ our study employs crystal lattice tailoring and defect modulation to engineer a Cr^3+^‐activated cubic spinel‐like material, where strategic introduction of Cr^3+^ into octahedral Al^3+^/Li^+^ sites drives efficient NIR‐ML through a non‐trap‐based mechanism (**Figure** **1**). Experimental and density functional theory (DFT) analyses reveal that Cr^3+^ doping induces local lattice distortions in [AlO_4_] units, activating adjacent O and Al sites to generate mid‐gap states that facilitate stress‐driven electron tunneling to Cr^3+^ luminescent centers, bypassing traditional trap‐recombination processes. The resulting narrow‐band emission at 711 nm (attributed to the spin‐forbidden ^2^E_g_ → ^4^A_2g_ transition) exhibits exceptional biological tissue penetrability compared to Mn^2+^‐based visible emitters, validated through proof‐of‐concept bio‐stress detection prototypes. Leveraging the dual luminescence of the visible host matrix and NIR Cr^3+^ centers, an advanced bright‐field anti‐counterfeiting model is demonstrated, merging spectral multiplexing with mechanical responsiveness. This work establishes a defect‐distortion coupling framework for self‐powered NIR‐ML, transcending conventional trap‐control paradigms and providing a design blueprint for next‐generation Cr^3+^‐based smart materials with integrated mechanical‐optical transduction capabilities for biomedical and security applications.

## 2 Results and Discussion

### 2.1 Structural Characterizations

**Figure 2:**
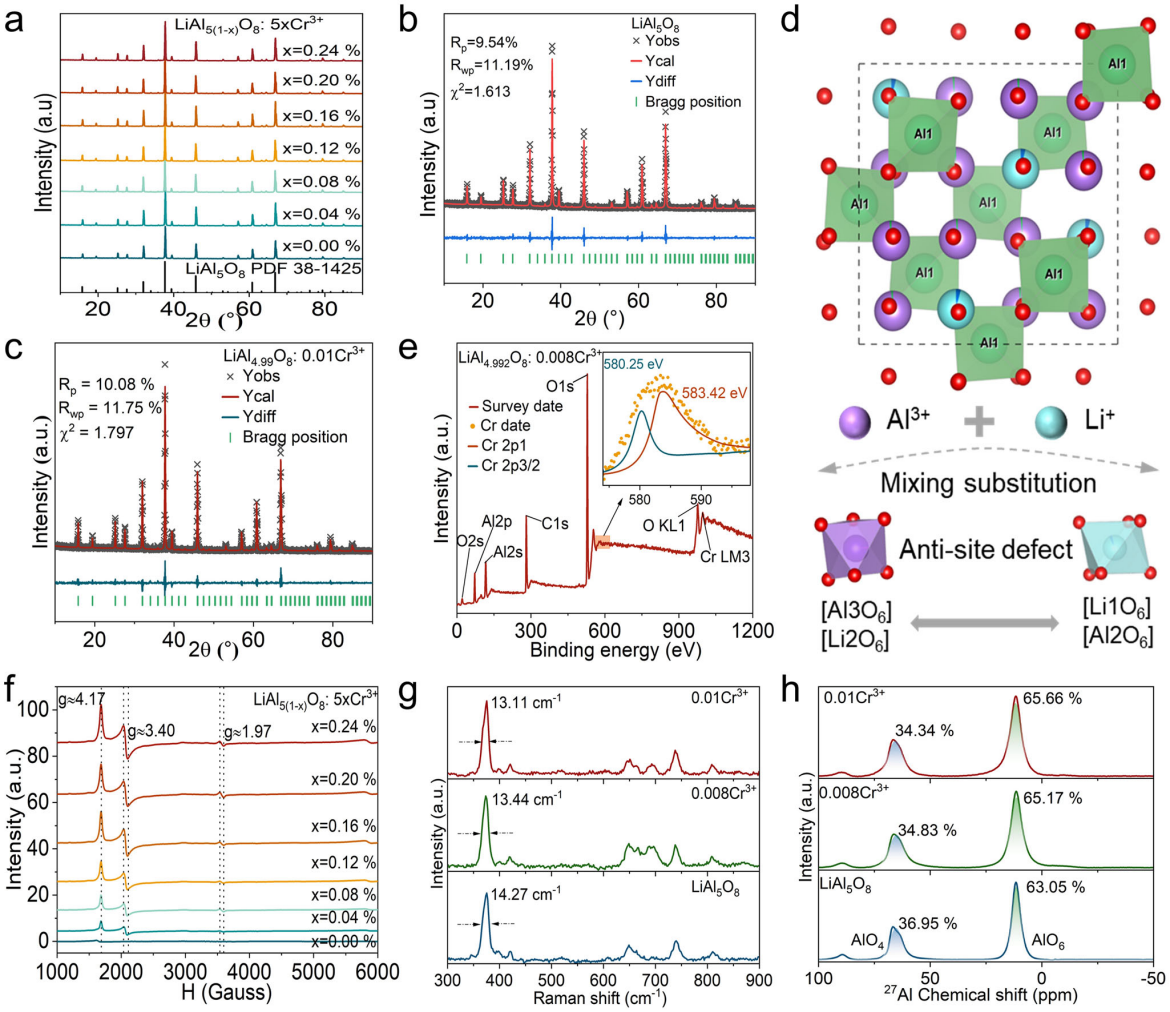
Structure Characterizations. a) XRD patterns, and b) Rietveld refinement of LiAl_5_O_8_. c) Rietveld refinement of LiAl_5(1‐x)_O_8_: 5xCr^3+^. d) Schematic diagram of LiAl_5_O_8_ structure with anti‐site defect formation. e) XPS survey and high revolution curve of LiAl_4.992_O_8_: 0.008Cr^3+^. f) EPR curves with corresponding g‐factor. g) Raman spectra of LiAl_5_O_8_: xCr^3+^ (x = 0, 0.008 and 0.01) phosphor. The arrows marked the FWHM of the main signal peaks. h) ^27^Al NMR spectra of LiAl_5_O_8_: xCr^3+^ (x = 0, 0.008 and 0.01) phosphor.

The atmospheric synthesis of LiAl_5(1‐x)_O_8_:5xCr^3+^ (x = 0.00% to 0.24%) yielded polycrystalline samples whose synchrotron XRD patterns exhibit excellent congruence with the cubic spinel LiAl_5_O_8_ reference (PDF 38–1425), devoid of secondary phase signatures (**Figure** **2**a). Compared to the x = 0.00% sample, GSAS‐based Rietveld refinement of the x = 0.20% sample (Rp = 10.08%, Rwp = 11.75%, χ^2^ = 1.797) corroborates structural homogeneity, with refined unit cell parameters (a = b = c ≈ 7.91 Å, V ≈ 495 Å^3^) marginally expanded relative to the undoped host‐attributed to isovalent Cr^3+^ (0.615 Å, coordination number (CN) = 6) substituting Al^3+^ (0.535 Å, CN = 6) with lattice distortion (Figure 2b,c; Table S1, Supporting Information). The P4332 (No. 212) space group assignment reveals a complex spinel architecture featuring [Al_1_O_4_] tetrahedra and mixed [Li_1_O_6_]/[Li_2_O_6_]/[Al_2_O_6_]/[Al_3_O_6_] octahedra, arising from Li^+^‐Al^3+^ cation disorder during high‐temperature synthesis (Figure 2d). Given Cr^3+^ stability in octahedral coordination and ionic radius matching compared to Al^3+^ in octahedral sites, structural modeling confirms preferential occupation of octahedral Al^3+^ positions over tetrahedral sites, a conclusion reinforced by bond valence sum calculations and electron paramagnetic resonance spectroscopy (Figure S1, Supporting Information).

To further validate the substitution mechanism, the radius discrepancy between Cr^3+^ and host cations was systematically analyzed using Equation S1 (Supporting Information), revealing a 19.08% radius difference for Li^+^ (0.76 Å) versus 14.95% for Al^3+^ (0.535 Å) in octahedral coordination. This quantitative analysis confirms preferential substitution of Al^3+^ by Cr^3+^ (0.615 Å), though the modest numerical gap allows for limited Li^+^ site occupancy – an inference supported by the marginal unit cell expansion observed in XRD refinements. The spinel lattice's [Al_2_O_6_] and [Al_3_O_6_] octahedra exhibit distinct structural characteristics: [Al_2_O_6_] features uniform Al_2_‐O_1_ bond lengths (2.0418 Å), while [Al_3_O_6_] displays heterogeneous bonds (Al_3_─O_1_ = 1.8535 Å, Al_3_─O_2_ = 1.9508 Å, Al_3_─O_1_ = 1.9090 Å), yielding a Baur distortion index (D) of 0.16034 (Equation S2, Supporting Information). This structural asymmetry, characterized by non‐ideal O─Al_3_─O bond angles deviating from 180°, creates locally deformed crystal fields that enhance mechanical responsiveness, which is a critical factor for ML. Rietveld refinement of LiAl_4.99_O_8_:0.01Cr^3+^ (Table S2, Supporting Information) confirms preferential occupation of the more distorted [Al_3_O_6_] sites, aligning with energy minimization principles for transition metal ions.

Valence state characterization via XPS revealed core‐level peaks at 580.25 eV (Cr 2p_1_) and 583.42 eV (Cr 2p_3/2_) (Figure 2e), consistent with Cr^3+^ oxidation states.^[^ ^30^ ^]^ EPR spectroscopy at room temperature exhibited g‐values of 4.17 and 3.40, assigned to isolated Cr^3+^ ions per Landry theory,^[^ ^31^ ^]^ while a g ≈ 1.97 signal indicated first‐nearest‐neighbor ion pairs (Figure 2f),^[^ ^32^ ^]^ ruling out significant Cr^4+^ contributions. The substitution of Cr^3+^ for Al^3+^ ions in the octahedral site will lead to an increase in the distortion of the local crystal field. Hence, we use Raman and ^27^Al nuclear magnetic resonance (NMR) test methods to characterize the degree of distortion inside the LiAl_5_O_8_: Cr^3+^ host. The Raman band at 374 cm^−1^ is attributed to the asymmetric bending of the Li─O bond; the Raman band at 739 cm^−1^ is assigned to the Al─O stretching vibration of the AlO_4_ tetrahedron.^[^ ^33^ ^]^ As the Cr^3+^ doping concentration increases, the narrowing of the Raman spectra indicates the increase of local distortion (Figure 2g). Moreover, the two main Gaussian‐fitted signals at 11.6 and 66.4 ppm are attributed to AlO_6_ and AlO_4_, which originate from normally tetrahedral coordinated Al^3+^ ions and anti‐site/normally octahedral coordinated Al^3+^ ions in Li^+^ sites, respectively.^[^ ^34^ ^]^ Interestingly, the increased proportion of AlO_6_ indicates that the inversion degree is increased with increasing Cr^3+^ content, which further verifies the increase of distorted local structure (Figure 2h). SEM imaging of LiAl_4.992_O_8_:0.008Cr^3+^ revealed irregular massive particles with 0.5 µm average diameters, featuring rough surfaces that may enhance mechanical stress concentration (Figure S2, Supporting Information). EDS mapping confirmed homogeneous distribution of Cr, Al, and O, though Li remained undetected due to low atomic mass; gold signals originated from conductive coating during sample preparation, ensuring reliable SEM conductivity (Figure S2f, Supporting Information). These structural and compositional insights establish a foundation for understanding how lattice distortion and defect chemistry modulate self‐powered mechanoluminescent behavior in Cr^3+^‐doped spinels.

### 2.2 Photoluminescence (PL) and Self‐Powered ML

**Figure 3:**
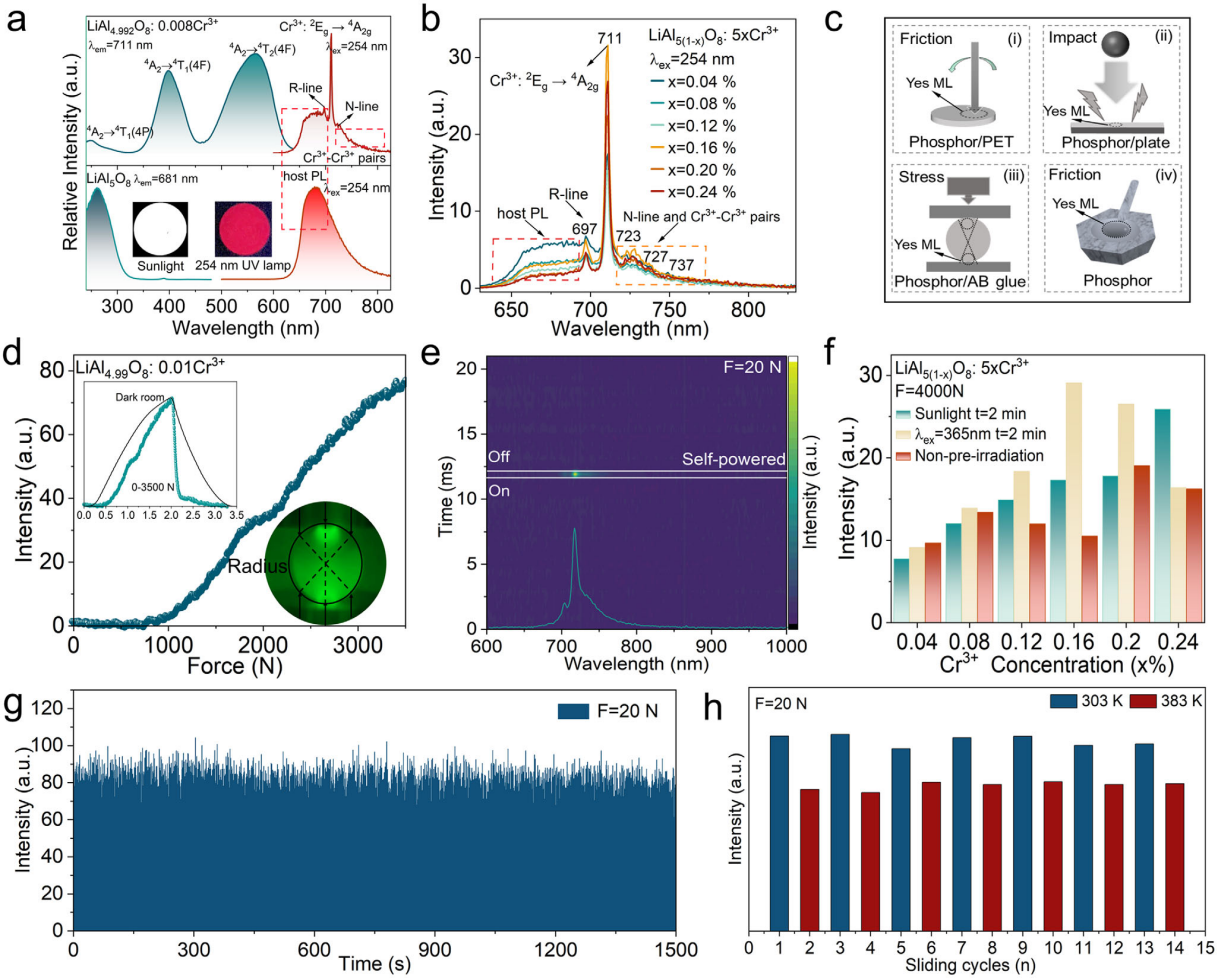
Luminescence Performance Characterizations. a) PL and PLE of LiAl_4.992_O_8_: 0.008Cr^3+^ when compared to the undoped LiAl_5_O_8_. b) PL spectra of LiAl_5(1‐x)_O_8_: 5xCr^3+^. c) Schematic diagrams of LiAl_5(1‐x)_O_8_: 5xCr^3+^ under different mechanical forces and non‐pre‐irradiation conditions, which includes 1) friction for LiAl_4.99_O_8_: 0.01Cr^3+^/PET, 2) impact for LiAl_4.99_O_8_: 0.01Cr^3+^ powder, 3) stress for LiAl_4.99_O_8_: 0.01Cr^3+^/AB glue, and 4) direct friction on LiAl_4.99_O_8_: 0.01Cr^3+^ powder. d) ML curve with external force (up to 3500 N) for LiAl_4.99_O_8_: 0.01Cr^3+^. Inset is the corresponding NIR‐ML image. e) 2D color map surface graphs of self‐powered ML recorded with time decay before and after force application under 20 N loading. f) Integrated ML intensity of LiAl_5(1‐x)_O_8_: 5xCr^3+^ (600–850 nm, 4000 N loading). g) Continuous stability and repeatability tests under F = 20 N. h) The fitted ML intensity with 20 N loading friction under 303 and 383 K.

The 711 nm emission in Cr^3+^‐doped LiAl_5_O_8_ arises from the spin‐forbidden ^2^E_g_ → ^4^A_2g_ transition of Cr^3+^ ions embedded in octahedral crystal fields, a phenomenon corroborated by Tanabe‐Sugano diagrams and spectral deconvolution.^[^ ^35^ ^]^ The luminescence spectrum exhibits fine‐structured features: a 697 nm zero phonon line (ZPL, R‐line) representing the unresolved electronic transition and a 723–737 nm band (N‐line) attributed to lattice vibrational coupling and Cr^3+^‐Cr^3+^ exchange interactions (**Figure** **3**a).^[^ ^36^, ^37^ ^]^ This spectral signature aligns with strong‐field coordination environments where crystal field splitting exceeds electron pairing energy, stabilizing the ^2^E_g_ excited state. The host contributes a broad 640–680 nm emission under 254 nm excitation, likely from oxygen vacancy defect states or Li^+^‐Al^3+^ anti‐site interactions, analogous to the luminescence mechanism observed in LiGa_5_O_8_ compounds. Photoluminescence excitation (PLE) spectra display three characteristic broad bands centered at 254, 399, and 564 nm, corresponding to ^4^A_2_ → ^4^T_1_(^4^P), ^4^A_2_ → ^4^T_1_(^4^F), and ^4^A_2_ → ^4^T_2_(^4^F) electronic transitions of Cr^3+^, respectively.^[^ ^38^ ^]^ Notably, PL emission spectra under different excitation wavelengths (252 , 399, and 564 nm) consistently feature peaks at 697, 711, 723 , 727, and 737 nm across all doping concentrations (x = 0.04%–0.24%), unequivocally confirming Cr^3+^ as the dominant luminescent center (Figure 3b; Figure S3, Supporting Information). The optimal PL intensity at x = 0.16% reflects a balance between Cr^3+^ site occupancy and concentration quenching, modulated by the disordered Al^3+^/Li^+^ cation distribution in the spinel lattice. Kubelka‐Munk analysis of diffuse reflectance spectra reveals a bandgap reduction from 4.31 eV (undoped host) to 4.17 eV in LiAl_4.992_O_8_:0.008Cr^3+^ (Equation S3, Supporting Information), attributed to the formation of O‐Cr hybrid states within the forbidden band (Figure S3h–I, Supporting Information).

In addition, we performed the temperature‐dependent photoluminescence and lifetime measurements from 100 to 500 K to further analyze phonon‐assisted decay mechanisms. As the temperature increased, the full width at half maximum (FWHM) of LiAl_5_O_8_: Cr^3+^ phosphor gradually increased, which can be ascribed to the strengthened electron–phonon interactions. Meanwhile, the red shift of the maximum PL peak is attributed to the change in the crystal field environment caused by the lattice thermal expansion (Figure S4a,b, Supporting Information). In addition, the relatively weak shift indicates that the rigid structure can inhibit the change. High structural rigidity can achieve good thermal stability.^[^ ^39^ ^]^ The integrated PL intensity and lifetime of LiAl_5_O_8_: Cr^3+^ phosphor decrease due to the non‐radiative transition (Figure S4c,d, Supporting Information). The electron is excited from the ground state to the excited state, then is thermally populated to the intersection of the ground state curve and the excited state curve, releasing the energy by way of non‐radiative relaxation.^[^ ^40^ ^]^ The strong coupling between Cr^3+^ ions and their surrounding crystal field significantly modulates the electronic structure of Cr^3+^, leading to distinct spectral emission characteristics that depend sensitively on local coordination environments. This phenomenon is most effectively visualized using the Tanabe‐Sugano (TS) diagram, a cornerstone of ligand field theory that maps d‐electron energy levels as a function of crystal field strength (*Dq*/B, where *Dq* is the crystal field splitting parameter and B is the Racah interelectronic repulsion parameter).^[^ ^41^, ^42^ ^]^ For Cr^3+^ (d^3^ configuration), the TS diagram predicts a critical transition from weak‐ to strong‐field regimes at *Dq*/B ≈ 2.3, a threshold that dictates whether the ion exhibits broadband (weak‐field) or narrowband (strong‐field) luminescence (Figure S4e, Supporting Information). When Cr^3+^ occupies octahedral sites in the LiAl_5_O_8_ lattice, the local crystal field strength can be quantitatively evaluated using Equation S4 (Supporting Information), which incorporates structural parameters such as metal‐ligand bond lengths and ligand electronegativity. This equation leverages the relationship between the crystal field splitting energy (Δo = 10*Dq*) and the geometric distortion of the [CrO_6_] octahedron, derived from the angular overlap model. For LiAl_5(1‐x)_O_8_: 5xCr^3+^, refinement of XRD data and bond valence sum calculations yield a *Dq*/B ratio of 2.39 within the strong‐field regime (*Dq*/B ≥ 2.3). This high crystal field strength suppresses non‐radiative relaxation pathways, stabilizing the spin‐forbidden ^2^E_g_ excited state and enabling the narrowband 711 nm emission observed in PL and ML spectra.^[^ ^43^ ^]^ The TS diagram not only rationalizes the observed narrowband emission but also provides a predictive framework for tailoring Cr^3+^ luminescence by engineering crystal field strength through lattice distortion or ligand substitution. Luminescence lifetimes (*τ*) (5.79–6.49 ms) follow single‐exponential decay kinetics, shortening with increasing Cr^3+^ concentration due to super‐exchange interactions between nearest‐neighbor ion pairs, as evidenced by EPR‐detected g≈1.97 signals (Figure S5a–c, Supporting Information).^[^ ^44^ ^]^

ML measurements demonstrate unprecedented self‐powered near‐infrared emission under diverse mechanical stimuli. When embedded in a PET composite, rubbed by a glass sleeve, impacted by an iron ball, or compressed by a universal testing machine, the material emits intense NIR light detectable via fiber‐optic spectroscopy (Figure 3c, Supporting Videos S1–S3, Supporting Information). A representative compression test on LiAl_5(1‐x)_O_8_: 5xCr^3+^/resin cylinders shows a linear increase in ML intensity with applied force (up to 3500 N) (Figure 3d). In addition, it shows a rapid 90% intensity decay within 0.1 s of force removal‐indicative of instantaneous stress‐optical transduction, which is ideal for dynamic sensing^[^ ^45^ ^]^ (Figure 3e). The ML is dominated by the spin‐forbidden ^2^E_g_ → ^4^A_2g_ transition, while no PersL emission is observed during the ML (Figure S5d–f, Supporting Information). The ML spectrum exhibits a minor red shift relative to PL, attributed to enhanced crystal field splitting under mechanical strain, which lowers the ^2^E_g_ energy level and redshifts the emission (Figure 1). Thermoluminescence (TL) measurements reveal no characteristic peaks across all doping concentrations, confirming that electron traps (0.60‐0.75 eV depth) do not contribute to PersL emission. Notably, the LiAl_5(1‐x)_O_8_: 5xCr^3+^ phosphors exhibit exceptional stress‐responsive autonomous NIR‐ML under a high compressive load of 4000 N without any external pre‐irradiation (Figure 3f; Figure S6, Supporting Information). This non‐pre‐irradiated ML performance contrasts starkly with conventional trap‐controlled systems, which require auxiliary light sources to activate luminescence. The ML intensity under pre‐irradiation (365 nm UV for 2 min) exceeds non‐irradiated conditions. However, this enhancement arises from minor contributions of intrinsic defects (V_O_, Al‐Li anti‐sites) rather than trap‐mediated processes (Figure S7, Supporting Information).

Force‐response characterization demonstrates exceptional linearity between mechanical input and ML output. Compression tests (2500–5000 N) yield a linear fit with R^2^ = 0.9976, while friction measurements (3–15 N) show R^2^ = 0.99673, validating the material as a high‐precision stress sensor (Figure S8, Supporting Information). Real‐time ML monitoring during cyclic friction tests reveals no signal degradation over 1500 cycles, underscoring its mechanical durability (Figure 3g, Supporting Video S4, Supporting Information). Real‐time ML spectra under cyclic friction were recorded via a fiber optic spectrometer (Figure S9a, Supporting Video S4, Supporting Information). The material's stability is critical for practical applications: PL intensity remains 70.31% of the initial value at 190 °C (Figure S9b, Supporting Information), demonstrating excellent thermal stability. Under cyclic friction tests at different temperatures, we found that the self‐powered ML intensity gradually decayed with increasing temperature, just like the PL, which is due to the electron–phonon interactions (Figure S9c–f, Supporting Information). These results highlight the material's robust self‐powered performance across diverse operational conditions (Figure 3h).

### 2.3 Self‐Powered NIR‐ML Mechanism

**Figure 4:**
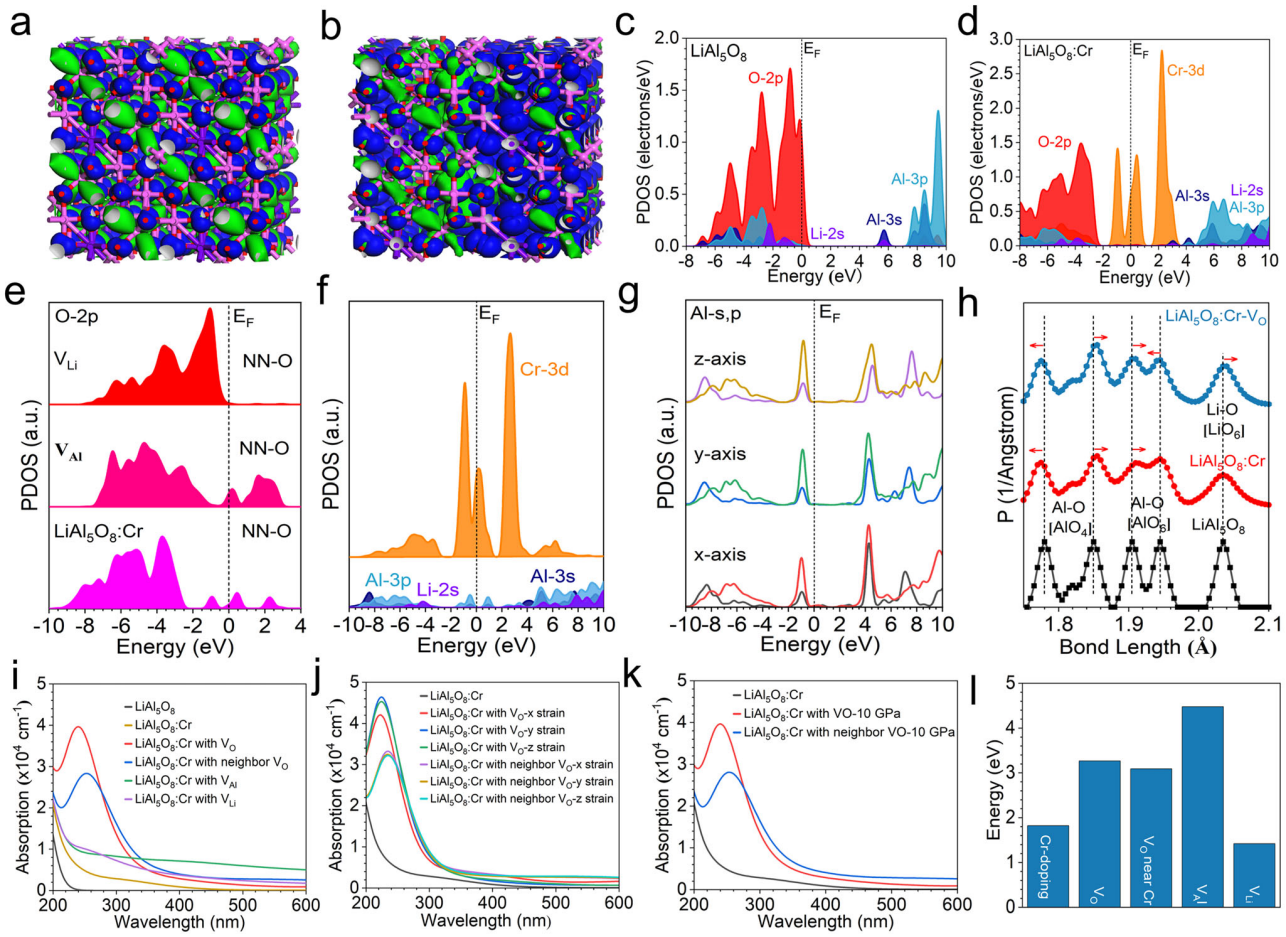
Theoretical calculations of LiAl_5_O_8_: Cr. Electronic distributions near the E_F_ in a) host LiAl_5_O_8_ and b) Cr‐doped LiAl_5_O_8_. Grey: Cr, Purple: Li, Pink: Al, Red: O. The bonding and anti‐bonding orbitals are demonstrated through blue iso‐surface and green iso‐surface, respectively. Electronic structure analysis of c) host LiAl_5_O_8_ and d) Cr‐doped LiAl_5_O_8_. e) Electronic structure analysis of the O‐2p orbitals of the NN‐O sites near different intrinsic defects. f) Electronic structure analysis of the NN‐sites near the V_O_. g) Electronic structure analysis of NN‐Al sites of Cr‐doping after introducing external stress from x,y, and z directions. h) The detailed bond length evolutions. i) The simulated absorption spectra of Cr‐doped LiAl_5_O_8_ with intrinsic vacancies. j) The simulated absorption spectra of Cr‐doped LiAl_5_O_8_ with Vo after introducing external stresses from (100), (010), and (001) directions. k) With the introduction of an external stress of 10 GPa, the simulated absorption spectra of Cr‐doped LiAl_5_O_8_ with Vo. (l) The energy costs of intrinsic defect formation.

Our prior investigations revealed that LiGa_5_O_8_:Cr^3+^ exhibits dual‐mode ML‐both self‐powered NIR emission and trap‐controlled PersL‐whereas isostructural LiGa_5_O_8_:Cr^3+^ displays exclusive self‐powered ML without PersL.^[^ ^29^ ^]^ This disparity motivated DFT studies to decode the underlying electronic mechanisms. The ordered bonding/antibonding orbital distribution near the Fermi level (E_F_) contrasts sharply with LiGa_5_O_8_, where Ga^3+^ (larger ionic radius than Al^3+^) induces lattice distortions that stabilize defect traps (**Figure** **4**a,b). For pristine LiAl_5_O_8_, electronic structure calculations through the projected density of states (PDOS) show a well‐defined bandgap of ≈4.50 eV, with valence band maximum (VBM) dominated by O‐2p orbitals and conduction band minimum (CBM) anchored by Al‐3p states; Li‐2s orbitals contribute minimally to the band edges (Figure 4c). Cr^3+^ doping in LiAl_5_O_8_ perturbs the electronic landscape: Cr‐3d orbitals hybridize strongly with adjacent O‐2p and Al‐3p states, introducing mid‐gap states that cross E_F_ (Figure 4d). This hybridization lowers the bandgap to ≈4.17 eV, aligning with experimental Kubelka‐Munk results, and creates efficient electron transfer pathways from defect sites to Cr^3+^ luminescent centers. Notably, the absence of PersL in LiAl_5_O_8_:Cr^3+^ correlates with shallower defect traps.

DFT results indicate that oxygen vacancies (V_O_) in LiAl_5_O_8_ produce trap depths <0.5 eV, which are insufficient for thermal activation of PersL (Figure 4e). With the existence of defects, the comparative PDOS analyses of the nearest neighboring (NN) sites are also performed to show the electronic structure evolutions. Near the lithium vacancies (V_Li_), O‐2p orbitals remain unperturbed, whereas aluminum vacancies (V_Al_) induce unoccupied states above the Fermi level in neighboring O‐2p orbitals, acting as intermediate trapping states. Critically, Cr‐doping activates the NN‐O sites, generating mid‐gap states that align with Cr‐3d orbitals, enabling efficient electron tunneling to luminescent centers. V_O_ similarly restructures adjacent Al‐3s/3p orbitals, creating gap states near E_F_ that overlap with Cr‐3d levels, enhancing carrier coupling (Figure 4f). External strain applied along three crystallographic axes further modulates NN‐Al orbitals near Cr dopants, promoting strong peak intensities near E_F_ that facilitate electron transfer during self‐powered ML (Figure 4g). These results establish that Cr‐doped NN sites coupled with collectively generate abundant mid‐gap states, activating stress‐driven electron transitions without trap‐mediated processes. The synergistic defect‐strain engineering thus underscores the non‐trap mechanism for self‐powered NIR‐ML in LiAl_5_O_8_:Cr^3+^, distinguishing it from PersL‐prone systems.

Detailed structural analysis reveals that Cr^3+^ doping induces anisotropic bond modifications in LiAl_5_O_8_, triggering structural rearrangements critical for ML. In [AlO_4_] tetrahedra, Al─O bonds of 1.78 and 1.85 Å are shortened to 1.75 Å and elongated to 1.88 Å, respectively, increasing tetragonal distortion by 12% (Figure 4h). Conversely, [AlO_6_] octahedra exhibit minor bond adjustments (1.90→1.91 Å and 1.95→1.94 Å), reducing local asymmetry. This enhanced [AlO_4_] distortion activates a cooperative Jahn‐Teller effect, lowering the energy barrier for electron transitions. V_O_ adjacent to Cr dopants do not amplify lattice deformation, with bond length variations <0.02 Å, verifying that Cr^3+^ doping is the primary driver of structural changes. Further optical analyses show V_O_ introduces a distinct absorption peak at 245 nm, which is stronger than in pristine LiAl_5_O_8_, while V_Li_ and V_Al_ only marginally enhance absorption (Figure 4i). External strain along [010] and [001] axes redshifts this peak to 225 nm with evidently increased intensity, attributed to stress‐induced narrowing of the bandgap (Figure 4j). Under the 10 GPa compression, optical properties remain stable due to the rigid spinel framework, with mild absorption intensity fluctuating (Figure 4k). Defect formation energy calculations reveal V_Li_ has the lowest energy (0.72 eV), whereas V_O_ formation energy difference varies very limitedly across lattice sites (Figure 4l). The overall relatively high formation energy of most defects restricts their concentration, explaining the minor trap‐mediated contribution to ML observed experimentally.

### 2.4 Self‐Powered NIR‐ML for Potential Bio‐Application

**Figure 5:**
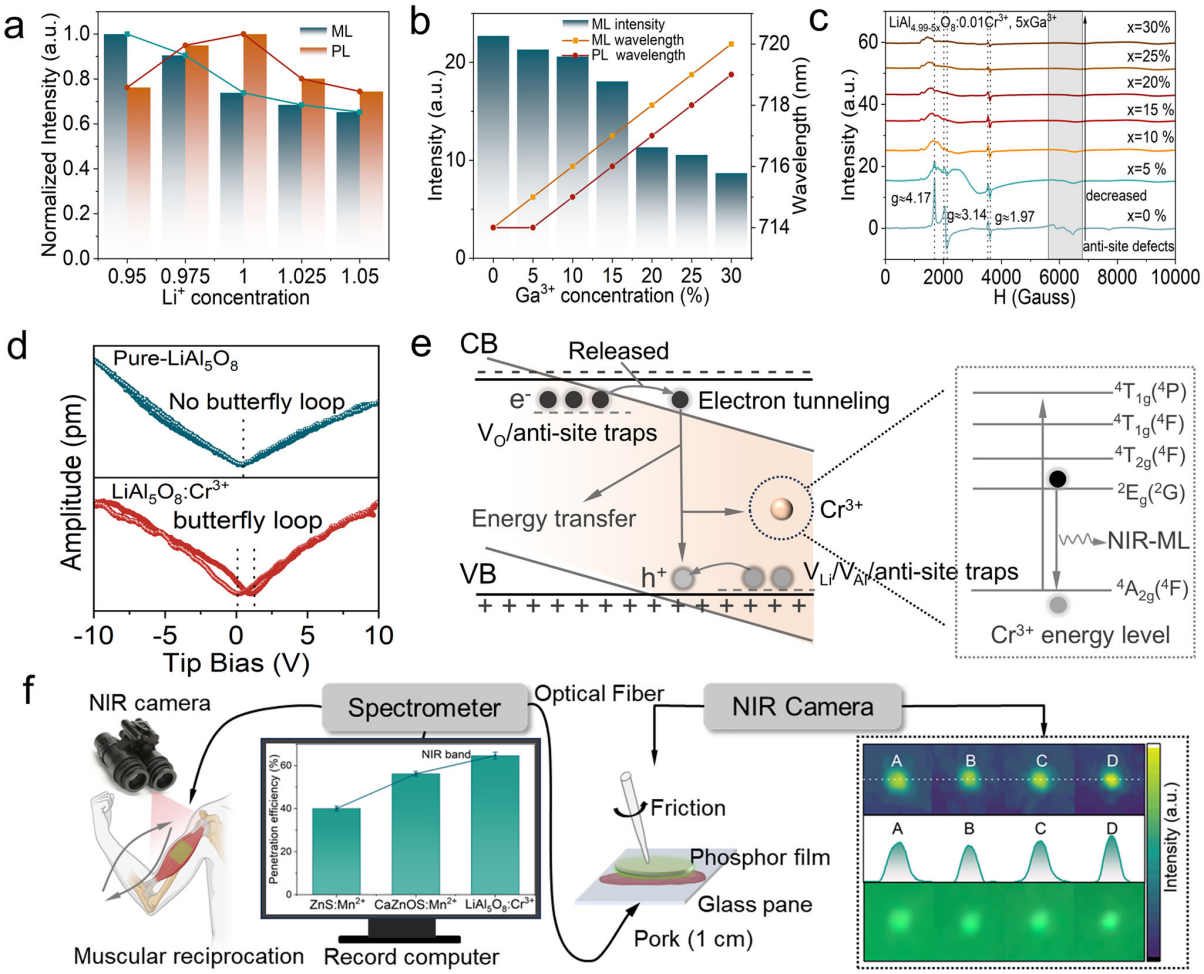
The potential bio‐application investigations. a) Normalized ML and PL intensity of Li_x_Al_4.99_O_8_:0.01Cr^3+^. The b) Integrated ML intensity, and ML and PL wavelength, and c) EPR curves with further introduction of Ga^3+^ in the Li_x_Al_4.99_O_8_:0.01Cr^3+^. d) PFM amplitude of pure and Cr^3+^‐doped LiAl_5_O_8_ host under a tip bias in the range of ±10 V, and after smoothing the curve. e) The schematic demonstration of the mechanism of the self‐powered ML in Li_x_Al_4.99_O_8_:0.01Cr^3+^. f) The penetration efficiency comparisons of LiAl_5_O_8_: Cr^3+^ with commonly reported ML materials (ZnS: Mn^2+^, CaZnOS: Mn^2+^) under the friction PET film. The proof‐of‐concept NIR biomechanical imaging scenario is also supplied.

To decode the defect‐driven self‐powered ML mechanism, lattice and defect engineering were employed to systematically modulate intrinsic distortions and defect concentrations in LiAl_5_O_8_: Cr^3+^.^[^ ^46^ ^]^ XRD patterns of Li_x_Al_4.99_O_8_: 0.01Cr^3+^ phosphors align with the standard LiAl_5_O_8_ host, confirming that artificial control of V_Li_ and Cr^3+^ doping does not induce phase transformations (Figure S10a, Supporting Information). EPR and Raman spectral features remain unchanged across samples, indicating minimal disruption to the crystal framework despite defect engineering (Figure S10b,c, Supporting Information). Regulating Li_2_O stoichiometry reveals that V_Li_ vacancies gradually transform into interstitial Li (i_Li_) as Li raw material decreases, concomitantly reducing V_O_ concentrations, which is consistent with prior theoretical calculations (Equation S6, Supporting Information). Artificially induced V_Li_ leads to lower PL intensity than stoichiometric samples, yet ML intensity correlates positively with V_Li_ concentration, demonstrating that V_Li_ facilitates ML emission (**Figure** **5**a; Figure S10d–f, Supporting Information). In addition, the formation of V_Li_ defects is due to the migration of Li atoms in high‐temperature solid‐state reactions. In order to further investigate the relationship between how defects affect emission kinetics, we performed a time‐resolved photoluminescence (TRPL) test. According to the TRPL test, there are multiple Cr^3+^ luminescence centers that contribute to PL emission (^2^E_g_ → ^4^A_2g_ transition, R‐line and N‐line), which is attributed to the defect altering the local symmetry^[^ ^47^ ^]^ (Figure S11a–c, Supporting Information). Notably, as the concentration of V_Li_ defects increases, the lifetime of the Cr^3+^ gradually increases, which is attributed to the fact that the defects change the local coordination environment of the Cr^3+^ ions to form a Cr^3+^‐defect recombination center, further inhibiting the originally fast non‐radiative transition^[^ ^38^ ^]^ (Figure S11d–f, Supporting Information). In general, the defects are no longer “quenching centers”, but reconstruct the Cr^3+^ excited state, causing the electron to quickly transition to the Cr state to produce the self‐powered ML. Lattice engineering via Ga^3+^ doping (with a larger ionic radius than Al^3+^) induces XRD peak shifts to lower angles due to unit cell expansion, while maintaining the cubic LiAl_5_O_8_ main phase (Figure S12a, Supporting Information). Raman spectra exhibit wavenumber downshifting attributed to the higher atomic weight of Ga^3+^ relative to Al^3+^ (Figure S12b, Supporting Information).^[^ ^48^, ^49^ ^]^ In Ga^3+^‐doped samples, luminescence lifetimes shorten with increasing Ga content, reflecting decreased *Dq* around Cr^3+^ (Figure S12c, Table S3, Supporting Information). Concomitantly, PL, PLE, and ML spectral peaks shift to longer wavelengths upon Al^3+^/Ga^3+^ co‐doping, as the crystal field intensity of [AlO_6_] exceeds that of [GaO_6_], influencing luminescent mechanisms (Figure 5b; Figure S11d,e, Supporting Information). Previous research indicates that anti‐site defects can be characterized via high‐field EPR, though specific experimental details align with the established framework of defect‐induced electronic modifications.^[^ ^50^, ^51^ ^]^ These results collectively demonstrate that defect engineering and lattice tuning synergistically optimize self‐powered ML by modulating charge transport pathways and crystal field environments, without introducing persistent luminescence interference.

As illustrated in Figure 5c, the concentration of anti‐site defects shows a decreasing trend with increasing Ga^3+^ doping, contrasting with the artificially controlled V_Li_ defects. This discrepancy arises because Ga^3+^ ions modulate the mixing degree between Ga^3+^/Al^3+^ and Li^+^ ions, thereby influencing anti‐site defect concentrations. Concomitantly, the ML intensity decreases in tandem with anti‐site defect reduction, highlighting that self‐powered ML stems from the collaborative action of multiple defects. The crystal field strength difference between Ga^3+^ and Al^3+^ ions drives the gradual redshift of ML and PL peak positions with increasing Ga^3+^ content. Notably, while local piezoelectricity is recognized to enable self‐powered ML in doped systems, its role in centrosymmetric cubic crystals like LiAl_5_O_8_ remains underexplored.^[^ ^52^ ^]^ Piezoelectric force microscopy (PFM) measurements reveal that pure LiAl_5_O_8_ exhibits negligible piezoelectricity, whereas Cr^3+^‐doped samples display distinct butterfly loops under a tip bias of −10 to 10 V (Figure 5d). This weak yet observable piezoelectricity originates from broken inversion symmetry induced by Cr^3+^ doping, which generates local piezoelectric fields. Integrating DFT calculations and experimental findings, a potential electron transfer model is proposed to explain the self‐powered ML mechanism (Figure 5e). Similar to PL, ML differs primarily in electron origin: lattice distortion induces local piezoelectric fields, generating polarization charge carriers that separate electron‐hole pairs under mechanical stress. Recombination of these carriers activates the Cr^3+^ luminescent center, with V_Li_, V_O_, and anti‐site defects near the valence/conduction band edges facilitating ML emission. Cr^3+^ doping introduces mid‐gap trap states, serving as intermediates for efficient electron tunneling to Cr sites, thus enabling robust self‐powered NIR‐ML.

The self‐powered NIR‐ML materials devoid of PersL offer a unique advantage by mitigating interference from ambient light sources, thereby enhancing the signal‐to‐noise ratio in ML imaging. This attribute positions them as promising candidates for high‐precision bright‐field anti‐counterfeiting, intelligent sensing, and biomedical detection. Building on the phosphor's demonstrated performance, proof‐of‐concept applications in bio‐stress monitoring and information security are herein proposed. A PET composite film was fabricated to enable functional characterization, showcasing distinct luminescent behaviors of QR‐code patterns under varying excitation sources when observed by the naked eye versus a NIR camera (Figure S13a,b, Supporting Information). The visible red QR‐code under 254 nm UV excitation originates from host matrix luminescence, while the NIR emission‐imperceptible to the naked eye, reveals hidden information (e.g., “SCUT”) under sunlight or 365 nm UV illumination when captured by an infrared camera, highlighting its utility in advanced anti‐counterfeiting technologies (Figure S13c, Supporting Information). The self‐powered operation of NIR‐ML, eliminating the need for pre‐irradiation, circumvents potential damage to biological tissues from external excitation sources. The 718 nm NIR narrowband emission exhibits superior tissue penetration compared to commercial Mn^2+^‐activated visible broadband phosphors (e.g., ZnS: Mn^2+^ at 590 nm and CaZnSO: Mn^2+^ at 620 nm) (Figure 5f). A proof‐of‐concept NIR biomechanical imaging model was established by aligning the composite film, biological tissue (pork), and fiber‐optic probe in a coaxial configuration (Figure S14, Supporting Information). Under frictional stimulation with a glass rod, NIR‐ML signals at ≈714 nm were detected through 10 mm of pork tissue, demonstrating excellent penetrability. The composite film's adaptability to muscle tissue surfaces enables real‐time NIR imaging of ML signals during mechanical deformation (rubbing/stretching), indicating its potential for in‐situ biomechanical sensing on human tissue interfaces. In summary, this self‐powered NIR‐ML phosphor integrates defect‐engineered lattice distortions and crystal field tuning to enable dual‐functionality: light‐source‐dependent information encryption and non‐invasive deep‐tissue bio‐detection.^[^ ^53^ ^]^ Its combination of mechanical energy harvesting and NIR optical specificity establishes a novel paradigm for next‐generation autonomous sensing systems, bridging the gap between materials science and biomedical technology.

## 3 Conclusion

This study presents the first demonstration of a self‐powered NIR‐ML material in Cr^3+^‐doped LiAl_5_O_8_, distinguished by its absence of persistent luminescence and operation without external pre‐irradiation‐unlike conventional trap‐controlled ML systems requiring auxiliary light sources. The narrow‐band NIR emission (711 nm) from octahedrally coordinated Cr^3+^ ions, confirmed via Tanabe‐Sugano diagrams, photoluminescence, and diffuse reflectance spectroscopy, enables unique stress‐responsive behavior validated through cyclic ML tests, showcasing remarkable stability and self‐recoverability for mechanical sensing. Mechanistic insights from lattice/defect engineering (Ga^3+^ doping, controlled Li vacancies) and density functional theory reveal that self‐powered ML arises from Cr^3+^‐induced local structural distortions, which activate neighboring Al and O sites to generate mid‐gap states for efficient electron tunneling to luminescent centers. Synergistic contributions from Li/Al/O vacancies and anti‐site defects further enhance carrier dynamics by modulating bandgap absorption. Leveraging the tissue‐penetration advantage of NIR emission, proof‐of‐concept biomedical applications are demonstrated, while the established defect‐distortion coupling model provides a transformative design paradigm for next‐generation self‐sustained ML materials, transcending traditional trap‐based mechanisms to enable advanced optomechanical transduction in smart sensing and bio‐imaging.

## Conflict of Interest

The authors declare no conflict of interest.

## Supporting information



Supporting Information

Supplemental Video 1

Supplemental Video 2

Supplemental Video 3

Supplemental Video 4

## Data Availability

The data that support the findings of this study are available from the corresponding author upon reasonable request.
