# Supplementary material for: Synergy between Defects and Lattice Distortion Drives Self‐Powered Elastico‐Near‐Infrared Mechanoluminescence in Cr3+‐Doped Spinel Oxides
Source: Adv Sci (Weinh). 2025 Aug 7;12(39):e10848. doi: 10.1002/advs.202510848 (PMC12533312; doi:10.1002/advs.202510848)
Supplement: Supplementary file 1 — Supporting Information [file ADVS-12-e10848-s005.docx]

Supporting Information

Synergy Between Defects and Lattice Distortion Drives Self-powered Elastico-Near-infrared Mechanoluminescence in Cr³⁺-doped Spinel Oxides

Yao Xiao^1,#^, Kang Chen^1,#^, Mingzi Sun^2,#^, Puxian Xiong^3,*^, Bolong Huang^2,*^, Yongsheng Sun^1^, Dongdan Chen^1^, Jiulin Gan^1,*^, and Zhongmin Yang^1,4^

**Experimental Section**

**Materials and syntheses**

The LiAl_5(1-x)_O_8_: 5xCr^3+^ (x = 0.00%, 0.04%, 0.08%, 0.12%, 0.16%, 0.20% and 0.24%) and all NIR-ML phosphors were prepared by the high-temperature solid reaction in air atmosphere. The raw materials include Cr_2_O_3_ (99.95%, Aladdin), Al_2_O_3_ (99.99%, Macklin), Ga_2_O_3_ (99.99%, Aladdin) and Li_2_CO_3_ (99.99%, Aladdin). All the raw materials were weighed according to certain stoichiometric ratios and thoroughly ground in a mortar, and then the corresponding obtained mixtures were sintered at 1350 °C for 8 h in alumina crucibles. Finally, samples were naturally cooled to room temperature and ground into powder for subsequent tests. To study the ML properties under loading force, the sample powders (1 g) were mixed with epoxy resin (4g) (A (1 g), B (3 g), type hard glue, DIY) to manufacture a cylinder with a thickness of 15 mm and diameter of 25 mm. The composited ML film (10 × 10 cm) was prepared by quickly molding the phosphor (2 g) with PET for friction ML test.

**Characterization Methods**

Powder sample XRD patterns were recorded by an X-ray diffractometer (Rigaku D/max-ⅢA) with CU-K_α1_ radiation (λ = 0.15405 nm; cathode voltage: 40/80 kV; current: 40 mA) in the 2θ range of 10 - 90° at room temperature. The refinement data and crystal structure parameters for LiAl_5(1-x)_O_8_: 5xCr^3+^ (x = 0.00%, 0.04%, 0.08%, 0.12%, 0.16%, 0.20% and 0.24%) samples were analyzed by using the GASA and Vesta software. Photoluminescence excitation (PLE), photoluminescence (PL) spectra and persistent luminescence (PersL) decay curves were recorded using the Edinburgh FLS920 Luminescence spectrometer equipped with a 450 W Xe lamp at room temperature. The optical resolution is maintained at an interval of 1 nm nanometer for data point acquisition. The luminescence lifetime was gained using a light source with 100 W μF900 lamp and a photo-multiplier detector. The Raman spectra were captured using a Raman spectrometer (Renishaw in Via, London, UK) with a 532 nm laser. The element distribution and surface morphology of LiAl_4.992_O_8_: 0.008Cr^3+^ sample was characterized by using a scanning electron microscopy (SEM, JSM-2010) equipped with an energy-dispersive X-ray spectrometer (EDS). X-ray photo-electron spectroscopy (XPS) was acquired using a model Axis Supra+ instrument (UK, Kratos Company). Electron paramagnetic resonance (EPR) measurements were collected using an EMXPLUS X-band spectrometer (Bruker, Germany). The diffuse reflectance spectra (DR) were obtained by a UV-Vis-NIR spectrophotometer (UH4150, Hitachi) over the spectral range of 250-800 nm (The optical resolution is set to 600). The thermoluminescence (TL) curves were recorded using a low temperature thermoluminescence three-dimensional spectrometer (LTTL-3DS-1) from 300 to 650 K range. Piezoelectric force microscopy (PFM) was carried out on an atomic force microscope (Oxford -MFP-3D Origin+). All ML spectra were recorded by a universal press machine (CMT1104), a photo-counting system (QE650 Pro, Ocean Optics), photomultiplier tube (PHOTOSENSOR MODULES H10722) and a computer in the dark condition. All NIR ML and PL images were obtained by an auxiliary night-vision monocular (ONV2+, Orpha, Germany).

**Calculation Setups**

To investigate the electronic structures of LiAl_5_O_8_: Cr, we have applied the density functional theory (DFT) calculations based on the CASTEP algorithm^[1]^. In particular, we have selected the generalized gradient approximation (GGA) and Perdew-Burke-Ernzerhof (PBE) functionals to offer accurate descriptions of the exchange-correlation interactions of the materials^[2]^. To guarantee the calculation accuracy, we have also applied the ultrasoft pseudopotentials for the plane-wave basis cutoff energy with ultrafine quality, which is 380 eV for LiAl_5_O_8_: Cr. Meanwhile, the Broyden-Fletcher-Goldfarb-Shannon (BFGS) algorithm has been utilized to achieve efficient energy minimizations, where the k-point settings have been set to 2×2×2 with coarse quality^[3]^. For the LiAl_5_O_8_: Cr, we have used a 1×2×2 supercell of LiAl_5_O_8_ by replacing one Al site with one Cr atom, which indicates a doping concentration of 2.5%. For all the geometry optimizations, we have applied stringent convergence criteria to guarantee calculation accuracy, where the Hellmann-Feynman forces should not exceed 0.001 eV/Å, the total energy difference should not be over 5×10^-5^ eV/atom, and the inter-ionic displacement should be less than 0.005 Å.


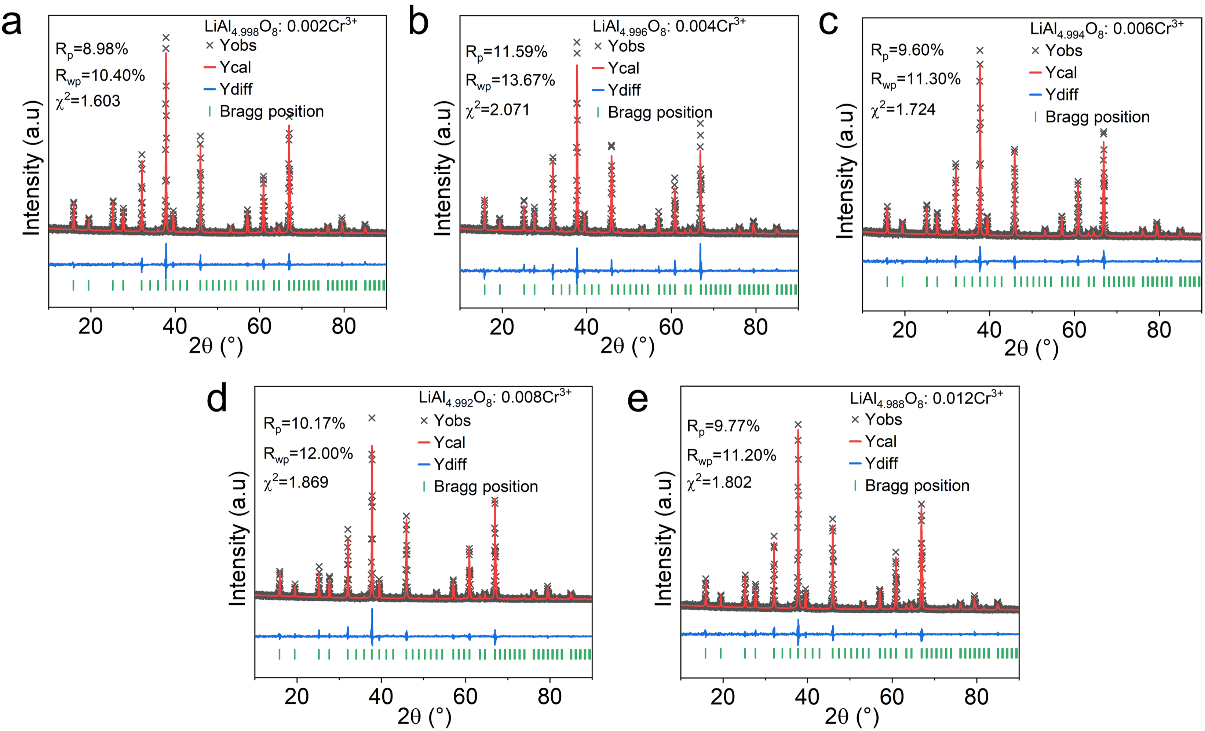


**Figure S1.** (a-e) Rietveld refinement results of LiAl_5(1-x)_O_8_: 5xCr^3+^ (x = 0.04%, 0.08%, 0.12%, 0.16%, and 0.24%).


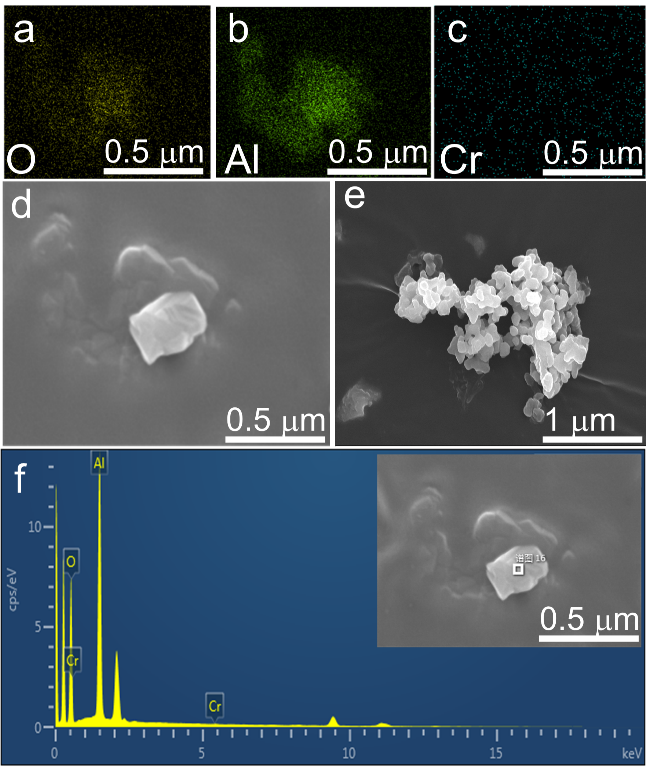


**Figure S2. (**a-c) The mapping images of elements O, Al, and Cr, respectively. (d-e) SEM images of LiAl_4.992_O_8_: 0.008Cr^3+^ at a scale of 0.5 μm (d) and 1 μm (f). EDS elements analysis selected by the area.


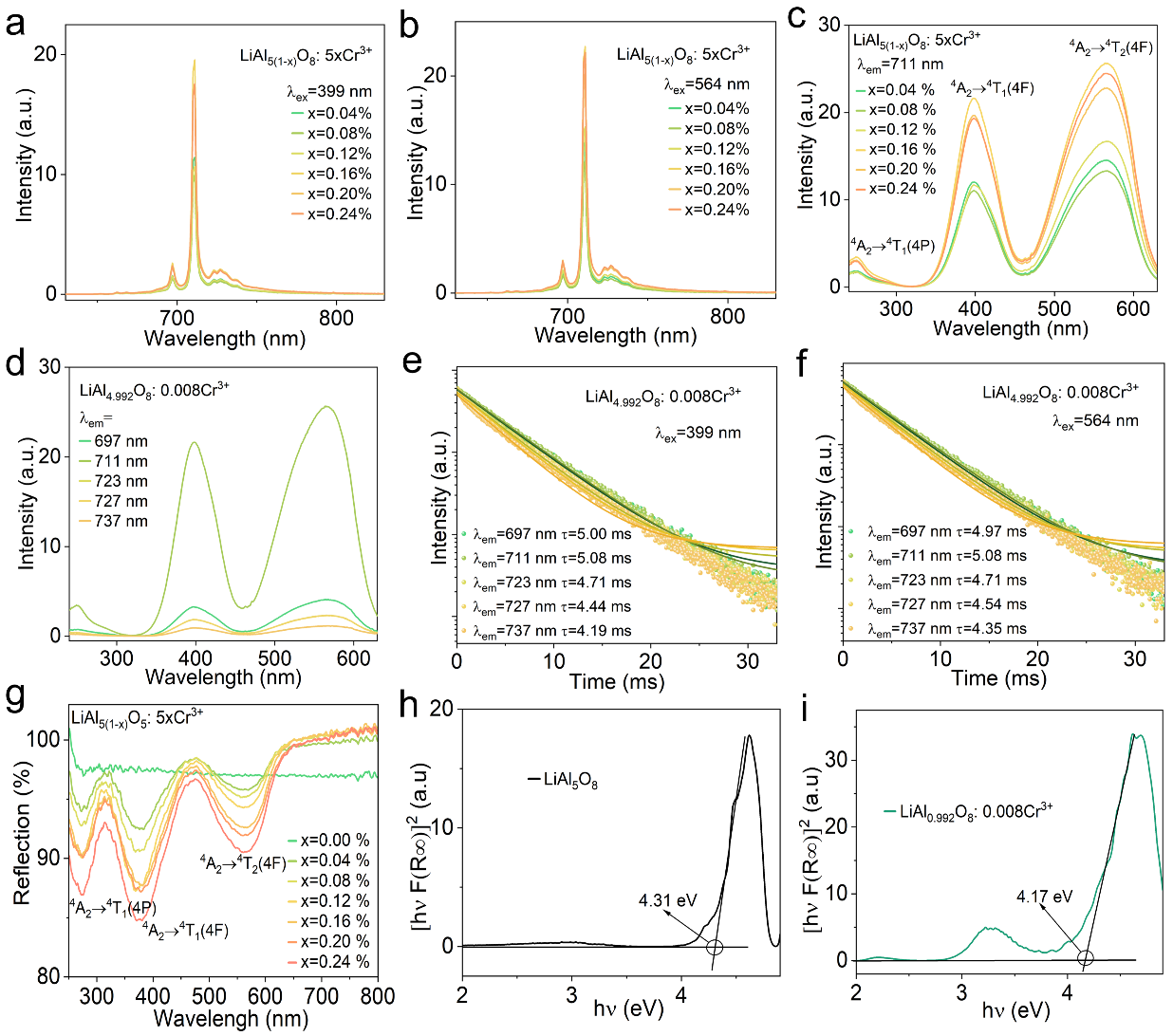


**Figure S3.** PL spectra of LiAl_5(1-x)_O_8_: 5xCr^3+^ (x = 0.04%, 0.08%, 0.12%, 0.16%, 0.20% and 0.24%) under excitation of (a) λ_ex_=399 nm, (b) λ_ex_=564 nm. (c) PLE spectra of LiAl_5(1-x)_O_8_: 5xCr^3+^ (x = 0.00%, 0.04%, 0.08%, 0.12%, 0.16%, 0.20% and 0.24%). (d) PLE spectra of LiAl_4.992_O_8_: 0.008Cr^3+^ monitored at λ_em_=697, 711, 723, 727, 737 nm. Luminescence lifetime decays of LiAl_4.992_O_8_: 0.008Cr^3+^under (e) λ_ex_= 399 nm, λ_em_=697, 711, 723, 727, 737 nm, respectively, (f) λ_ex_= 564 nm, λ_em_=697, 711, 723, 727, 737 nm, respectively. (g) DR spectra of LiAl_5(1-x)_O_8_: 5xCr^3+^ (x = 0.00%, 0.04%, 0.08%, 0.12%, 0.16%, 0.20% and 0.24%). Functional curve of [hv F(∞)]^2^ versus hv for calculation E_g_ value for (h) LiAl_5_O_8_ host, (i) LiAl_4.992_O_8_: 0.008Cr^3+^.


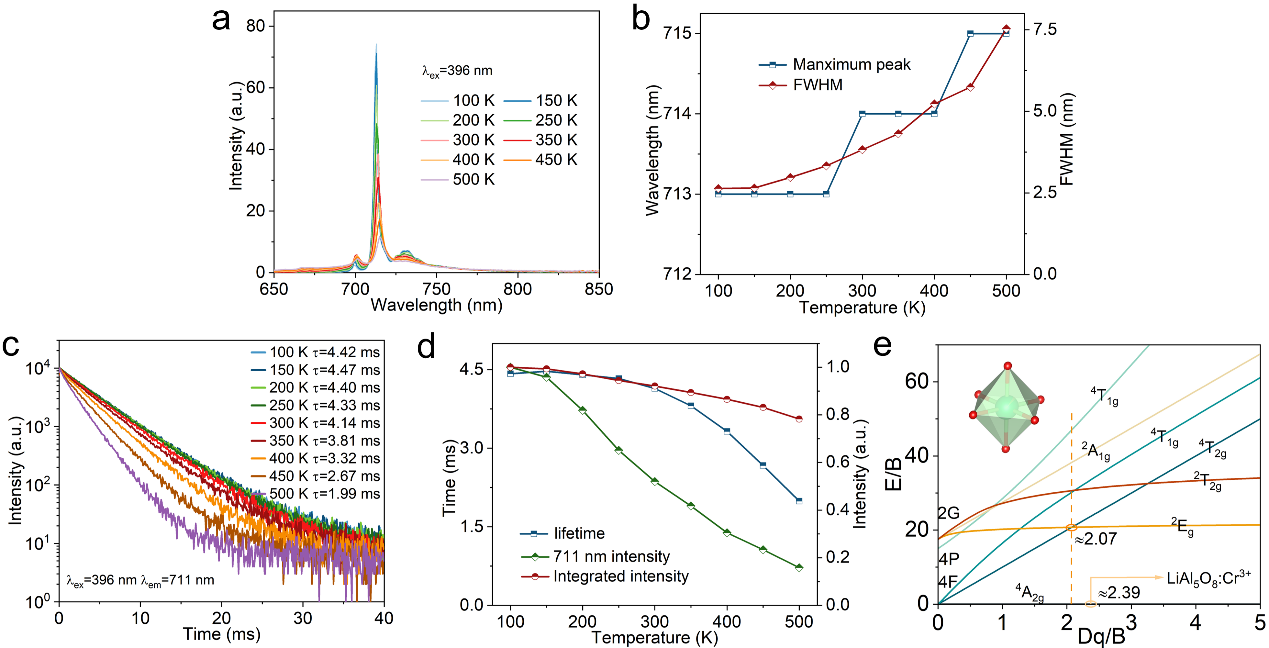


**Figure S4.** (a) The temperature-dependent PL spectra of LiAl_5_O_8_: 0.01Cr^3+^ phosphor from 100 to 500 K. (b) The relationship of PL maximum peak and FWHM with temperature changes. (c) The temperature-dependent luminescence lifetime decays. (d) The integrated PL intensity and average decay lifetime. (e) Tanabe-Sugano diagram of Cr^3+^ ion (3d^5^ electronic configuration).


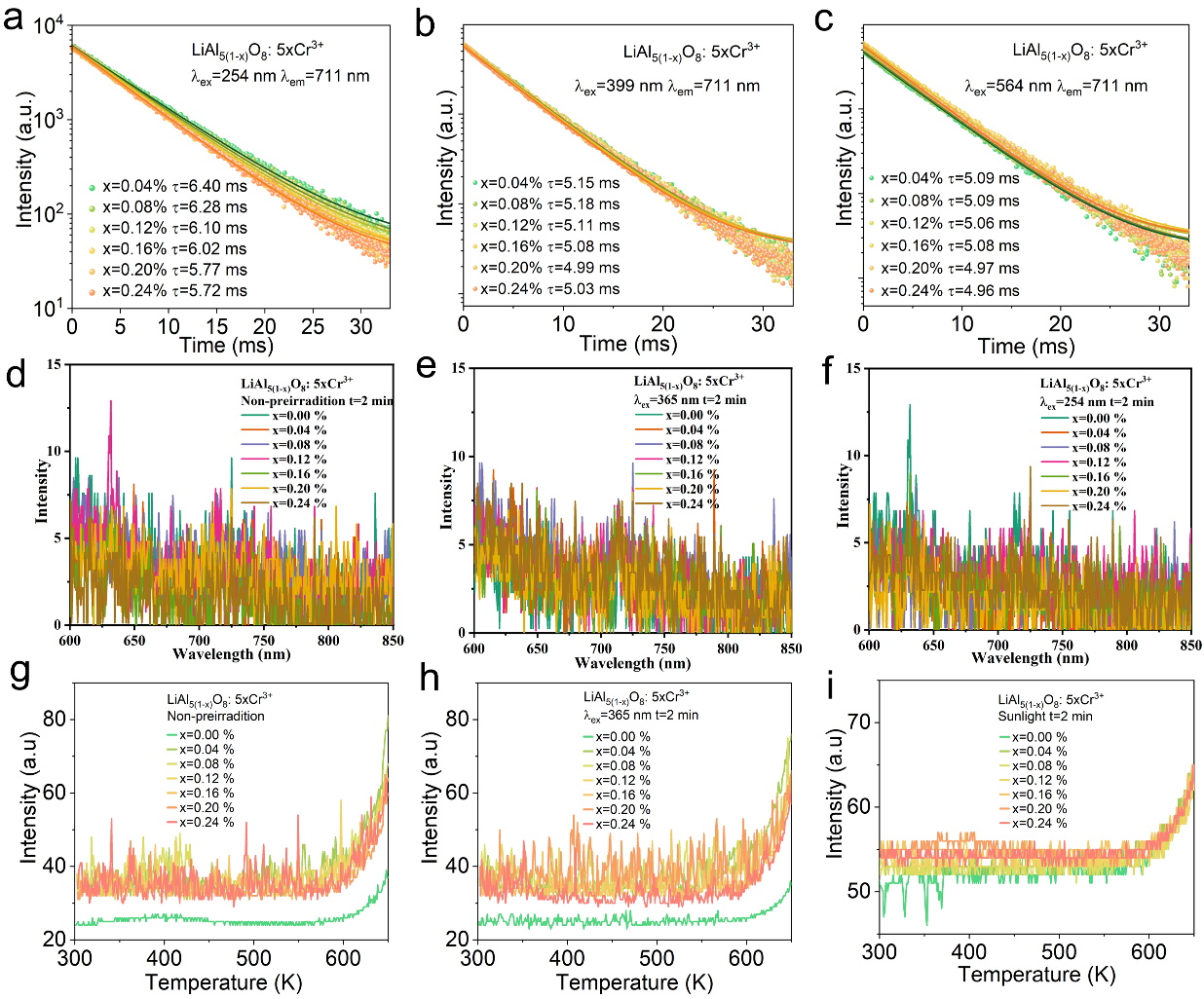


**Figure S****5.** Luminescence lifetime decays of LiAl_5(1-x)_O_8_: 5xCr^3+^ (x = 0.04%, 0.08%, 0.12%, 0.16%, 0.20% and 0.24%) under (a) λ_ex_=254 nm, λ_em_=711 nm, (b) λ_ex_=399 nm, λ_em_=711 nm, (c) λ_ex_=564 nm, λ_em_=711 nm. PersL and TL curves of LiAl_5(1-x)_O_8_: 5xCr^3+^ (x = 0.00%, 0.04%, 0.08%, 0.12%, 0.16%, 0.20% and 0.24%) under different excitation condition (d, g) Non-pre-irradiation, (e, h) 365 nm UV lamp for 2 min, (f, i) Sunlight for 2 min.


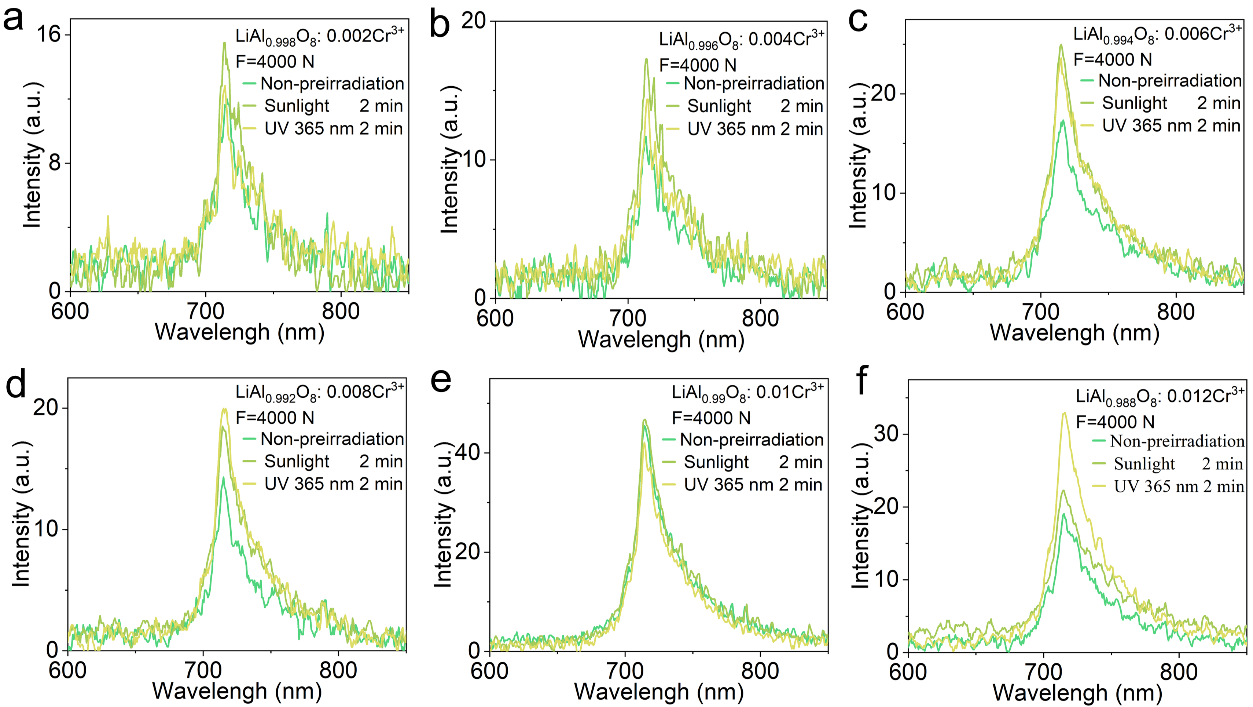


**Figure S6.** (a-f) ML spectra of LiAl_5(1-x)_O_8_: 5xCr^3+^ (x = 0.04%, 0.08%, 0.12%, 0.16%, 0.20% and 0.24%) under 4000 N loading and different excitation conditions (Non-pre-irradiation, Sunlight, and 365 nm UV lamp).


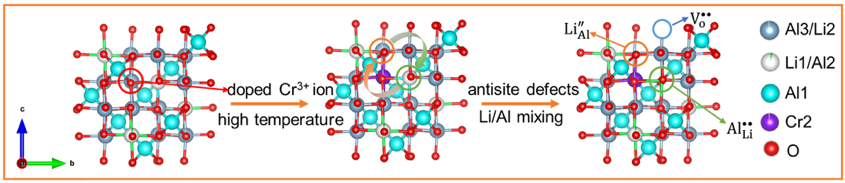


**Figure S7.** The formation process of an anti-site defect.


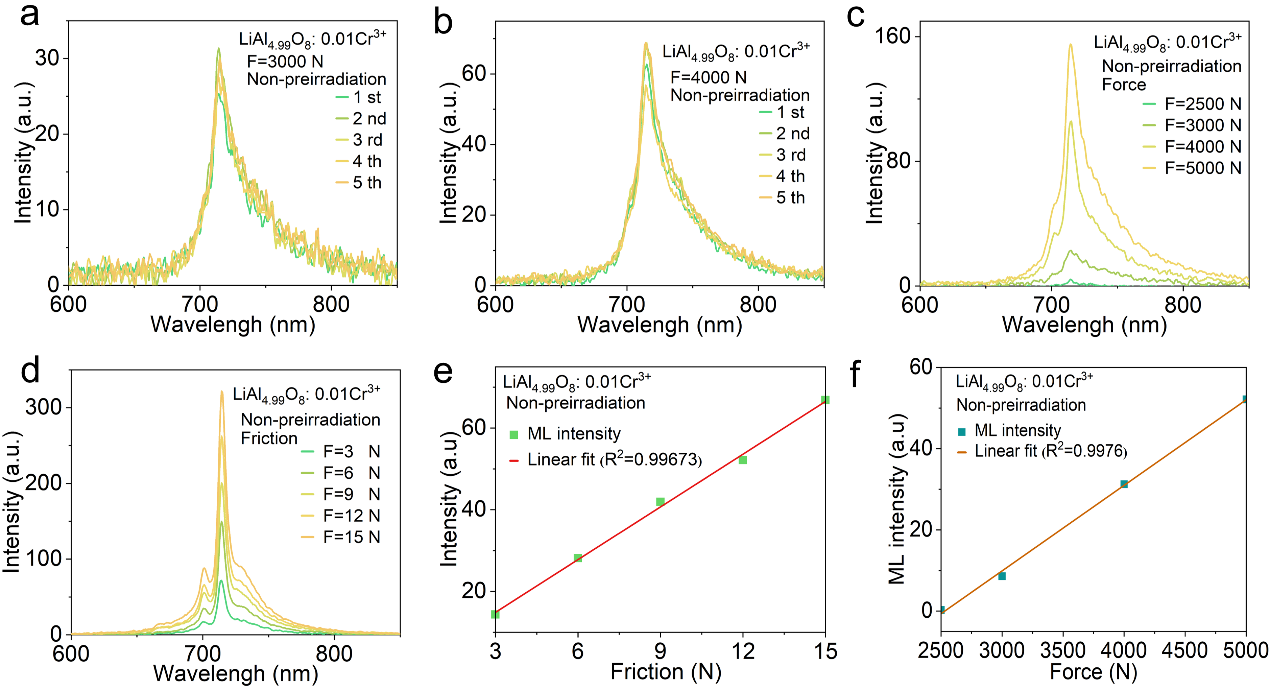


**Figure S8.** (a) ML spectra of LiAl_4.99_O_8_: 0.01Cr^3+^ recorded by five times at 2 min intervals under 3000 N loading and non-pre-irradiation. (b) ML spectra of LiAl_4.99_O_8_: 0.01Cr^3+^ recorded by five times at 2 min intervals under 4000 N loading and non-pre-irradiation (c) ML spectra of LiAl_4.99_O_8_: 0.01Cr^3+^ under different loading forces (2500 N, 3000 N, 4000 N, 5000 N). (d) ML spectra of LiAl_4.99_O_8_: 0.01Cr^3+^ under different loading friction (3 N, 6 N, 9 N, 12 N, 15 N). (e) ML spectra of LiAl_4.99_O_8_: 0.01Cr^3+^ as a function of loading friction (3 N, 6 N, 9 N, 12 N, 15 N). (f) ML spectra of LiAl_4.99_O_8_: 0.01Cr^3+^ as a function of loading force (2500 N, 3000 N, 4000 N, 5000 N).


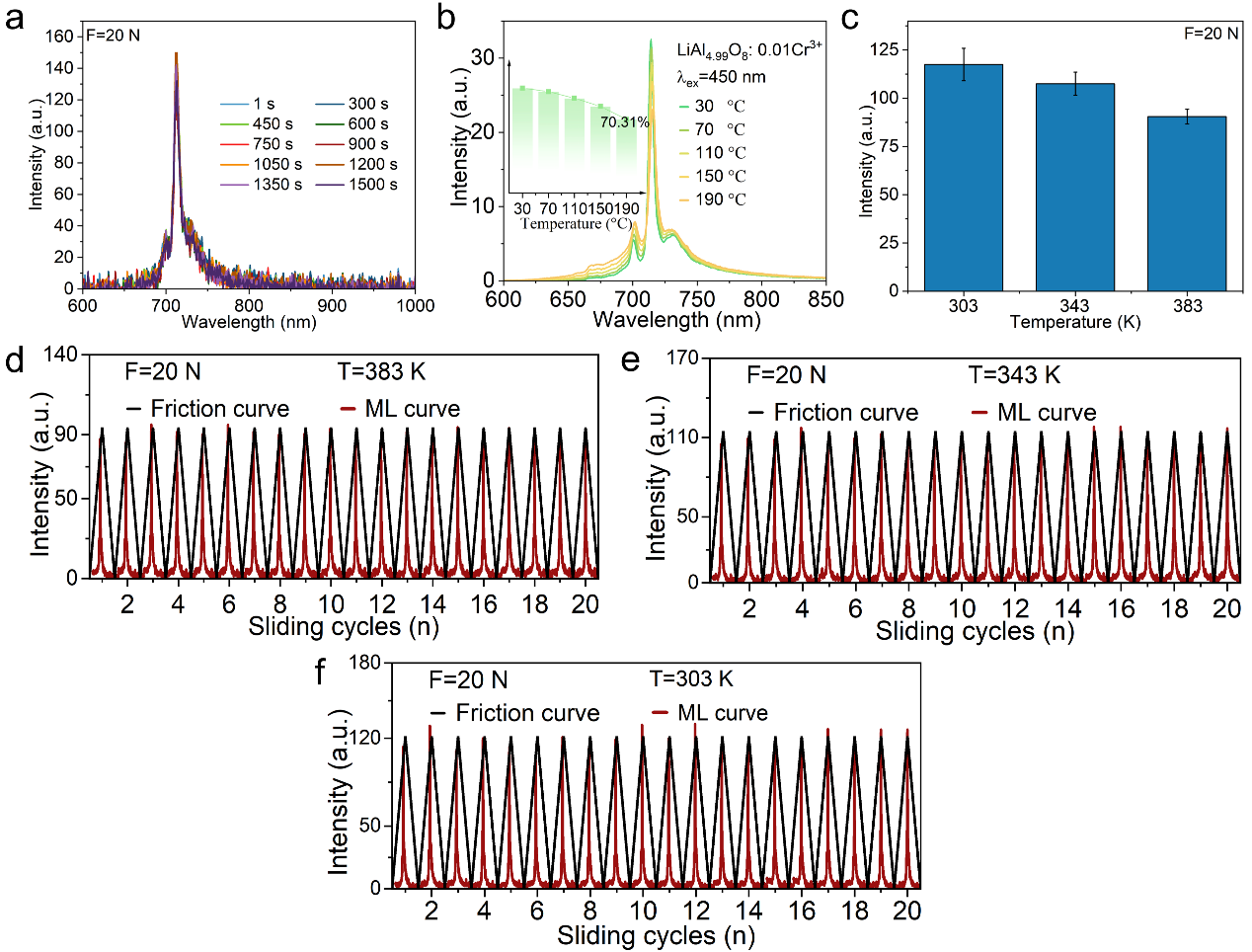


**Figure S9.** (a) The self-powered ML spectra under different recorded times. (b) PL spectra of LiAl_4.99_O_8_: 0.01Cr^3+^ under different temperatures (30, 70, 110, 150, 190 °C). (c) The integrated self-powered ML intensity under different temperatures with 20 N loading friction. The fitted ML intensity with 20 N loading friction under (d) 383 K, (e) 343 K, and (f) 303 K temperatures.


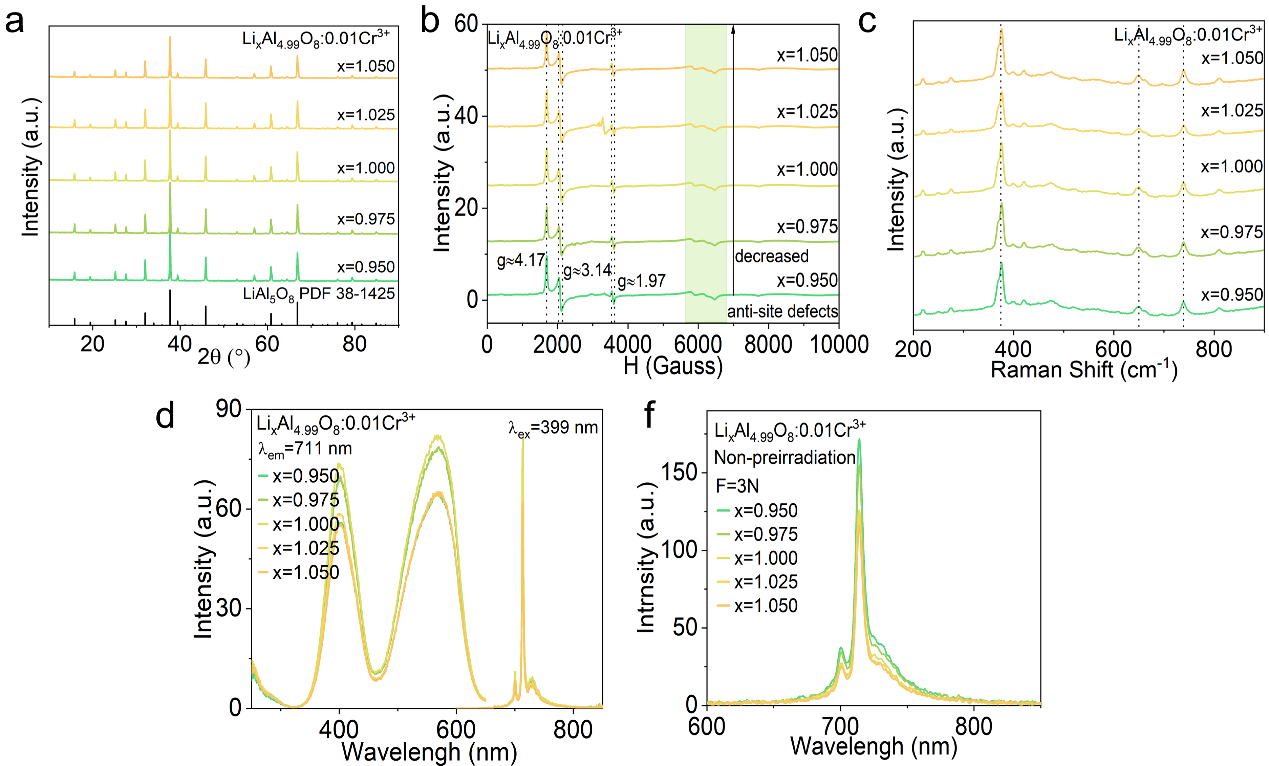


**Figure S10.** (a) XRD patterns; (b) EPR curves (c) Raman spectra; (d) PLE and PL spectra; (e) ML spectra of Li_x_Al_4.99_O_8_:0.01Cr^3+^ (x = 0.950, 0.975, 1.000, 1.025, 1.050)


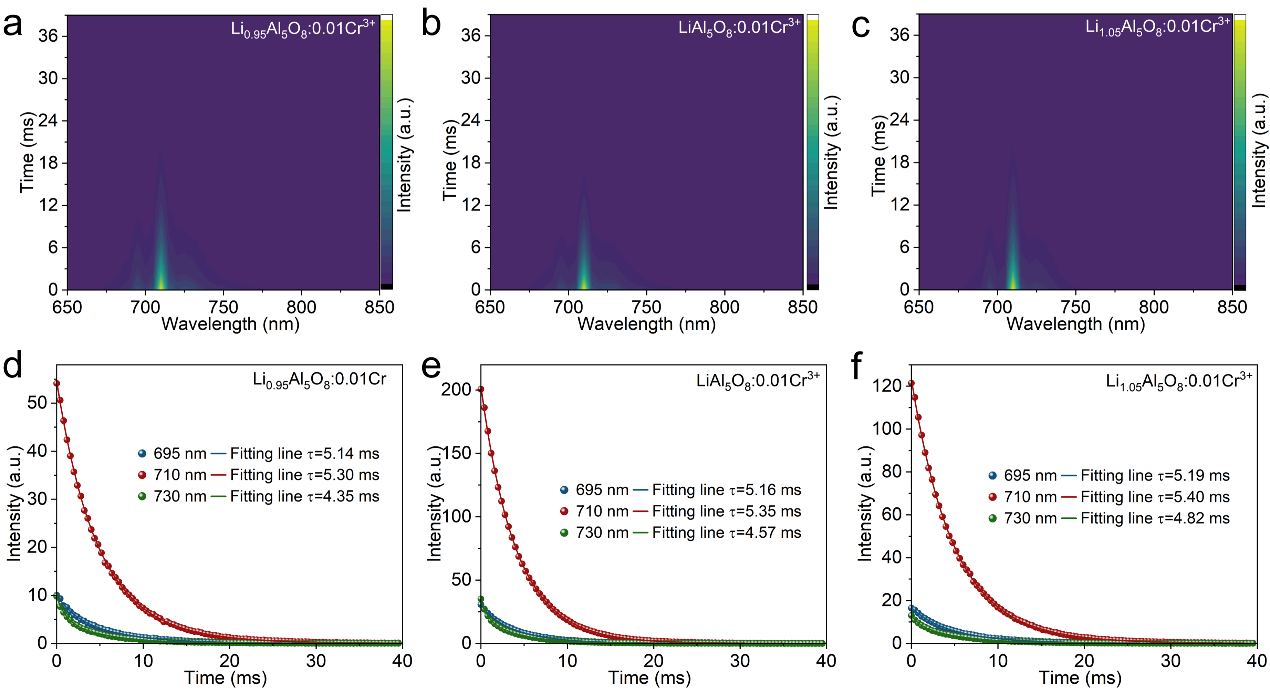


**Figure S11.** The 2D time-resolved PL spectra and the corresponding lifetime decay curve. (a, d) Li_0.95_Al_4.99_O_8_:0.01Cr^3+^. (b, e) LiAl_4.99_O_8_:0.01Cr^3+^. (c, f) Li_1.05_Al_4.99_O_8_:0.01Cr^3+^.


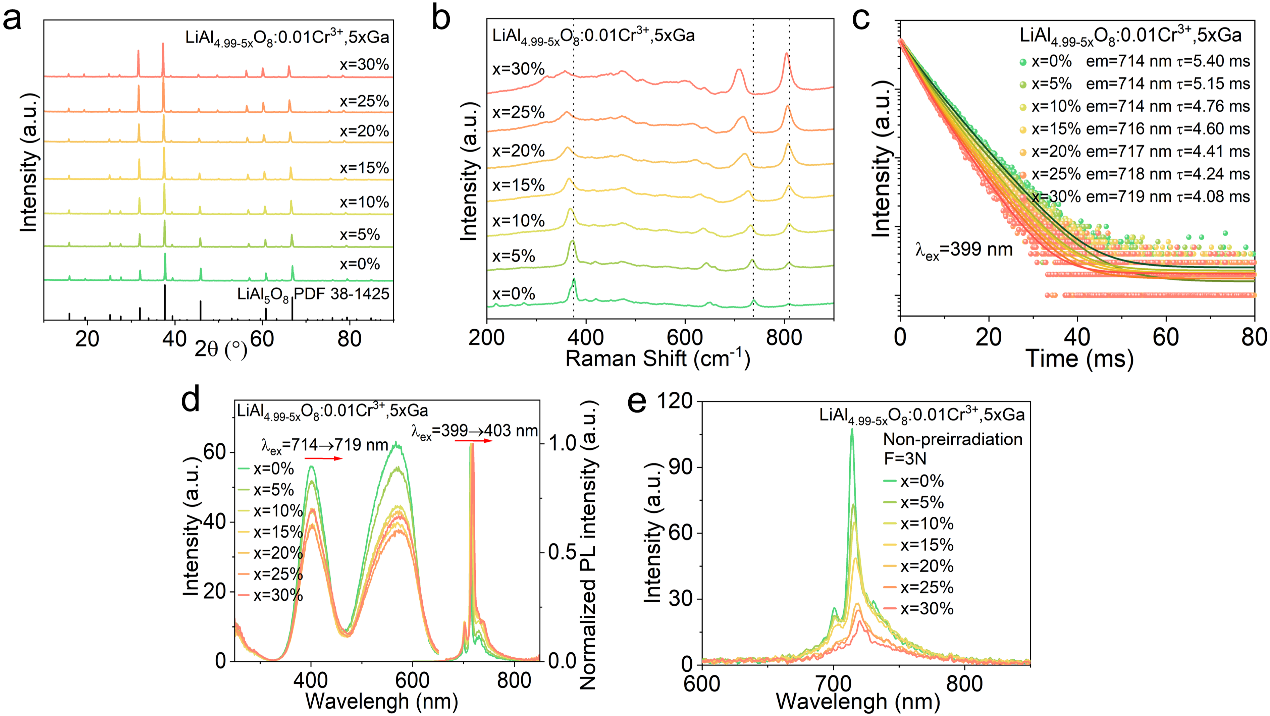


**Figure S12.** (a) XRD patterns; (b) Raman spectra; (c) Luminescence lifetime decays; (d) PLE and PL spectra; (e) ML spectra of LiAl_4.99-5x_O_8_: 0.01Cr^3+^,5xGa^3+^ (x = 0%, 5%, 10%, 15%, 20%, 25% and 30%).


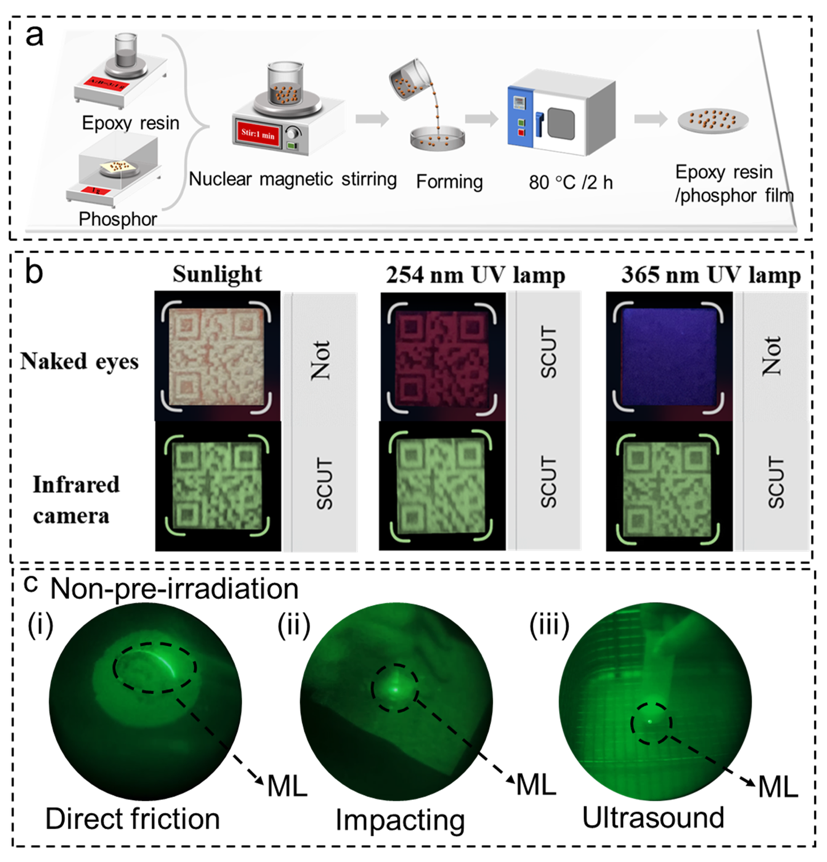


**Figure S13.** (a) Fabrication process of composite film by mixing ML powders and Epoxy resin. (b) PersL QR-code images under different pre-irradiation conditions. (c) ML images under different external stimuli.


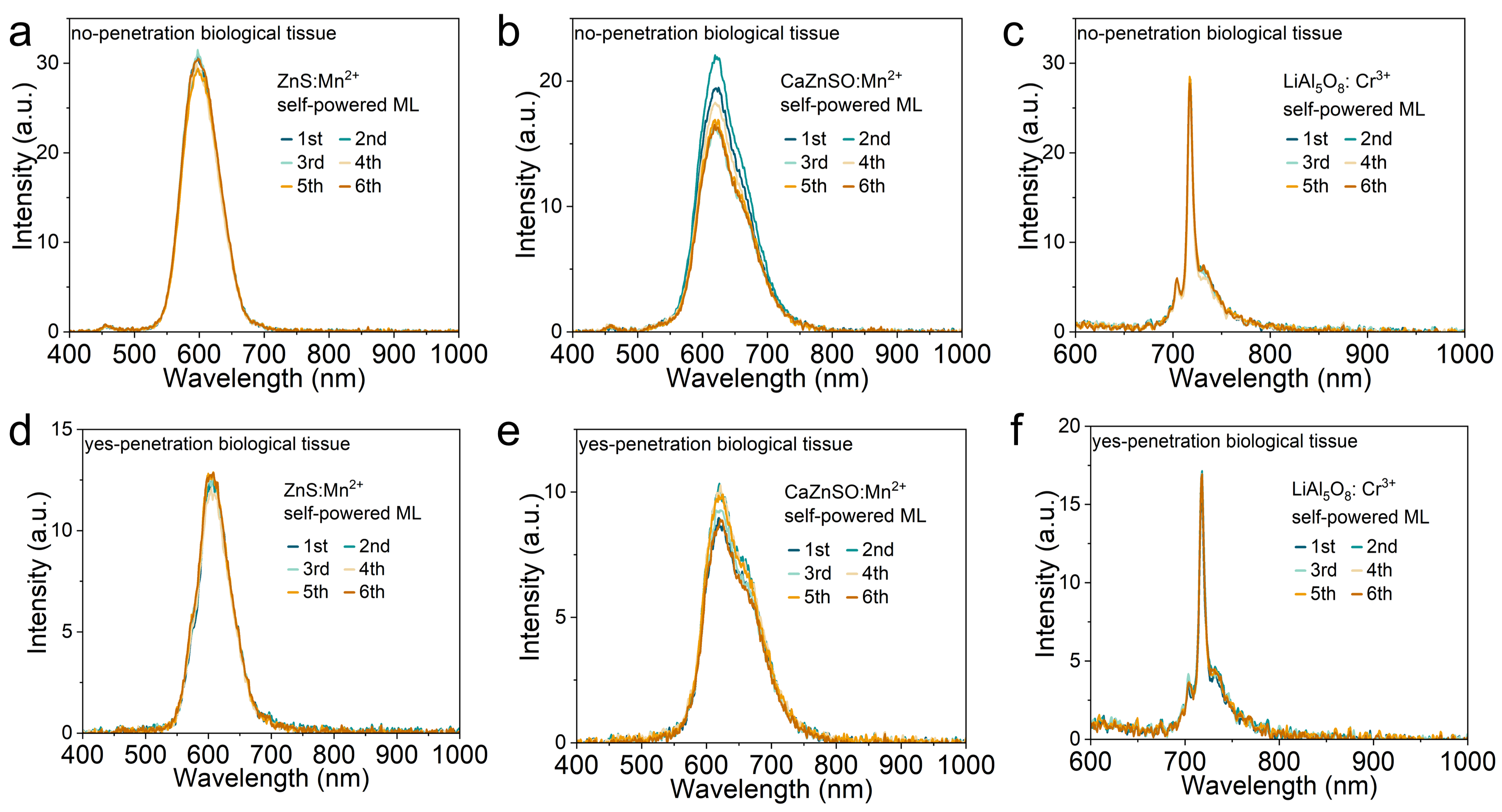


**Figure S14.** (a-c) The ML spectra of continued 20 N loading friction under no penetration biological tissue for ZnS: Mn^2+^, CaZnSO: Mn^2+^, and LiAl_5_O_8_: Cr^3+^ phosphor. (d-f) The ML spectra of continued 20 N loading friction under penetration (1 cm) biological tissue for ZnS: Mn^2+^, CaZnSO: Mn^2+^, and LiAl_5_O_8_: Cr^3+^ phosphor.

**Equation S1** Heme-Rothery empirical formula:

$$Dr=\frac{[|R_{m}(CN)-R_{d}(CN)|]}{R_{m}(CN)}$$

Where *R_m_* and *R_d_* are the Shannon ionic radii of the host substituted ion and dopant ion, respectively.

Equation S2

$\text{D=}\frac{1}{n}\sum_{i=1}^{n} \frac{\left|l_{i}-l_{av}\right|}{l_{av}}$

Where *l_i_* is the distance between the central atom and the *i* coordinated atom and *l_av_*

is the average bond length.

**Equation S3** Kubelka-Munk formula^[4]^:

$$[F(R_{\infty})h\mu)]^{^{n}}=K(h\mu-E_{_{g}})$$

Where *F(R_∞_) = (1-R_∞_)^2^/(2R_∞_)* originates from the Kubelka-Munk function, *R* is the reflectance (%), *n* = 2 for the LiAl_5_O_8_ host is proved direct-gap semiconductor material by the DFT calculation, *hμ* is the photon energy, *K* is constant and *E_g_* is the value of the bandgap.

**Equation S4** Crystal field parameters calculations^[5]^:

$$10Dq=E({}^{4}T_{2})=E({}^{4}A_{2}\to{}^{4}T_{2})$$

$$\frac{Dq}{B}=\frac{15(\Delta E/Dq-8)}{(\Delta E/Dq)^{2}-10(\Delta E/Dq)}$$

$$\Delta E=E({}^{4}T_{1})-E({}^{4}T_{2})=E({}^{4}A_{2}\to{}^{4}T_{1})-E({}^{4}A_{2}\to{}^{4}T_{2})$$

Where *Dq* is the crystal field strength, *B* is Racah constants, *E(^4^T_2_)* and *E(^4^T_1_)* represent the *^4^T_2_* and *^4^T_1_* energy levels, *E(^4^A_2_→^4^T_2_)* and *E(^4^A_2_→^4^T_1_)* is the transition energy from *^4^A_2_* ground state to the *^4^T_2_* and *^4^T_1_* excited state energy levels, respectively, which can be obtained by the PLE spectrum of LiAl_4.992_O_8_: 0.008Cr^3+^ sample.

**Equation S5** Luminescence lifetime calculated equation:

$$I(t)=I_{0}+A_{1}\exp(-\frac{t}{\tau})$$

Where *I(t)* is the Luminescence intensity, *A_1_* is a constant, *t* is time, and *τ* is the luminescence lifetime.

**Equation S6** Charge compensation between V_Li_ and Vo defect:

: Lack V_Li_ model

:

Enrich V_Li_ model

**Table S1** Rietveld refined crystallographic parameters of the LiAl_5(1-x)_O_8_: 5xCr^3+^ (x = 0.00%, 0.04%, 0.08%, 0.12%, 0.16%, 0.20% and 0.24%).

| Formula | x=0.00% | x=0.04% | x=0.08% | x=0.12% | x=0.16% | x=0.20% | x=0.24% |
| --- | --- | --- | --- | --- | --- | --- | --- |
| Crystal system | Cubic | Cubic | Cubic | Cubic | Cubic | Cubic | Cubic |
| Space group | P4332 | P4332 | P4332 | P4332 | P4332 | P4332 | P4332 |
| a (Å) | 7.9074 | 7.9073 | 7.9089 | 7.9085 | 7.9078 | 7.9087 | 7.9088 |
| b (Å) | 7.9074 | 7.90073 | 7.9089 | 7.9085 | 7.9078 | 7.9087 | 7.9088 |
| c (Å) | 7.9074 | 7.9073 | 7.9089 | 7.9085 | 7.9078 | 7.9087 | 7.9088 |
| α=β=γ (°) | 90 | 90 | 90 | 90 | 90 | 90 | 90 |
| volume (Å^3^) | 494.424 | 494.407 | 494.714 | 494.637 | 494.504 | 494.679 | 494.693 |
| Z | 4 | 4 | 4 | 4 | 4 | 4 | 4 |
| Density (g/cm^3^) | 3.625 | 3.625 | 3.626 | 3.623 | 3.624 | 3.627 | 3.623 |
| R_wp_ (%) | 11.19 | 10.40 | 13.67 | 11.30 | 12.00 | 11.75 | 11.20 |
| R_p_ (%) | 9.54 | 8.98 | 11.59 | 9.60 | 10.17 | 10.08 | 9.77 |
| χ^2^ | 1.613 | 1.603 | 2.071 | 1.724 | 1.869 | 1.797 | 1.802 |

**Table S2** Different atomic site occupancy and select interatomic angle of LiAl_4.99_O_8_: 0.01Cr^3+^. (The site of Al2, Li1 and Cr1 or Al3, Li2 and Cr2 is same, so that they have the same bond angle.)

| Element | x | y | z | Occupancy | Bond type | Bond angle (°) | |  |
| --- | --- | --- | --- | --- | --- | --- | --- | --- |
| l2 | 0.62500 | 0.62500 | 0.62500 | 0.0406 | Al2-O1 | |  | |
| Li1 | 0.62500 | 0.62500 | 0.62500 | 0.9570 | Al3-O1 | |  | |
| Cr1 | 0.62500 | 0.62500 | 0.62500 | 0.0024 | Al3-O2 | |  | |
| Al3 | 0.36860 | -0.11860 | 0.12500 | 0.9784 | O1-Al2-O1 | | 179.8996 | |
| Li2 | 0.36860 | -0.11860 | 0.12500 | 0.0140 | O1-Al2-O1 | | 179.8996 | |
| Cr2 | 0.36860 | -0.11860 | 0.12500 | 0.0076 | O1-Al2-O1 | | 179.8996 | |
| Al1 | -0.00190 | -0.00190 | -0.00190 | 1 | O1-Al3-O2 | | 174.7697 | |
| O1 | 0.11524 | 0.13297 | 0.38444 | 1 | O1-Al3-O1 | | 169.7441 | |
| O2 | 0.38481 | 0.38481 | 0.38481 | 1 | O2-Al3-O1 | | 174.7697 | |

**Table S3** crystal field strength Dq of the LiAl_4.99-5x_O_8_: 0.01Cr^3+^,5xGa^3+^ (x = 0%, 5%, 10%, 15%, 20%, 25% and 30%)

| LiAl_4.99-5x_O_8_:0.01Cr^3+^,5xGa^3+^ | ^4^A_2_→^4^T_1_ (nm) | | ^4^A_2_→^4^T_2_ (nm) | Dq/B |  |
| --- | --- | --- | --- | --- | --- |
| x=0 % | 402 | 567 | | 2.41478 | |
| x=5 % | 401 | 572 | | 2.29099 | |
| x=10 % | 403 | 576 | | 2.26972 | |
| x=15 % | 403 | 578 | | 2.23317 | |
| x=20 % | 402 | 577 | | 2.22529 | |
| x=25 % | 402 | 578 | | 2.20728 | |
| x=30 % | 402 | 579 | | 2.18942 | |

**References**

[1] S. J. Clark, M. D. Segall, C. J. Pickard, P. J. Hasnip, M. J. Probert, K. Refson, M. C. Payne, *Zeitschrift Fur Kristallographie* **2005**, 220, 567.

[2] a)J. P. Perdew, K. Burke, M. Ernzerhof, *Phys. Rev. Lett.* **1996**, 77, 3865; b)P. J. Hasnip, C. J. Pickard, *Comput. Phys. Commun.* **2006**, 174, 24; c)J. P. Perdew, J. A. Chevary, S. H. Vosko, K. A. Jackson, M. R. Pederson, D. J. Singh, C. Fiolhais, *Physical Review B* **1992**, 46, 6671.

[3] J. D. Head, M. C. Zerner, *Chem. Phys. Lett.* **1985**, 122, 264.

[4] L. Guo, T. Wang, Q. Wang, W. Feng, Z. Li, S. Wang, P. Xia, F. Zhao, X. Yu, *Chem. Eng. J.* **2022**, 442, 136236.

[5] a)A. Zhang, Y. Liu, G. Liu, Z. Xia, *Chem. Mater.* **2022**, 34, 3006; b)Y. Tanabe, S. Sugano, *J. Phys. Soc. Jpn.* **1954**, 9, 766.
